# Labile assembly of a tardigrade protein induces biostasis

**DOI:** 10.1002/pro.4941

**Published:** 2024-03-19

**Authors:** S. Sanchez‐Martinez, K. Nguyen, S. Biswas, V. Nicholson, A. V. Romanyuk, J. Ramirez, S. Kc, A. Akter, C. Childs, E. K. Meese, E. T. Usher, G. M. Ginell, F. Yu, E. Gollub, M. Malferrari, F. Francia, G. Venturoli, E. W. Martin, F. Caporaletti, G. Giubertoni, S. Woutersen, S. Sukenik, D. N. Woolfson, A. S. Holehouse, T. C. Boothby

**Affiliations:** ^1^ Department of Molecular Biology University of Wyoming Laramie Wyoming USA; ^2^ School of Chemistry University of Bristol Bristol UK; ^3^ Max Planck‐Bristol Centre for Minimal Biology University of Bristol Bristol UK; ^4^ Department of Biochemistry and Molecular Biophysics Washington University School of Medicine St. Louis Missouri USA; ^5^ Center for Biomolecular Condensates Washington University in St. Louis St. Louis Missouri USA; ^6^ Quantitative Systems Biology Program University of California Merced Merced California USA; ^7^ Department of Chemistry and Biochemistry University of California Merced Merced California USA; ^8^ Dipartimento di Chimica “Giacomo Ciamician” Università di Bologna Bologna Italy; ^9^ Laboratorio di Biochimica e Biofisica Molecolare, Dipartimento di Farmacia e Biotecnologie, FaBiT Università di Bologna Bologna Italy; ^10^ Consorzio Nazionale Interuniversitario per le Scienze Fisiche della Materia (CNISM), c/o Dipartimento di Fisica e Astronomia (DIFA) Università di Bologna Bologna Italy; ^11^ Department of Structural Biology St. Jude Children's Research Hospital Memphis Tennessee USA; ^12^ Van't Hoff Institute for Molecular Sciences University of Amsterdam Amsterdam The Netherlands; ^13^ School of Biochemistry University of Bristol, Biomedical Sciences Building Bristol UK

**Keywords:** anhydrobiosis, biomolecular condensation, desiccation tolerance, filament formation, gelation, intrinsically disordered protein, osmotic stress, tardigrade

## Abstract

Tardigrades are microscopic animals that survive desiccation by inducing biostasis. To survive drying tardigrades rely on intrinsically disordered CAHS proteins, which also function to prevent perturbations induced by drying in vitro and in heterologous systems. CAHS proteins have been shown to form gels both in vitro and in vivo, which has been speculated to be linked to their protective capacity. However, the sequence features and mechanisms underlying gel formation and the necessity of gelation for protection have not been demonstrated. Here we report a mechanism of fibrillization and gelation for CAHS D similar to that of intermediate filament assembly. We show that in vitro, gelation restricts molecular motion, immobilizing and protecting labile material from the harmful effects of drying. In vivo, we observe that CAHS D forms fibrillar networks during osmotic stress. Fibrillar networking of CAHS D improves survival of osmotically shocked cells. We observe two emergent properties associated with fibrillization; (i) prevention of cell volume change and (ii) reduction of metabolic activity during osmotic shock. We find that there is no significant correlation between maintenance of cell volume and survival, while there is a significant correlation between reduced metabolism and survival. Importantly, CAHS D's fibrillar network formation is reversible and metabolic rates return to control levels after CAHS fibers are resolved. This work provides insights into how tardigrades induce reversible biostasis through the self‐assembly of labile CAHS gels.

## 1 INTRODUCTION

Water is required for all metabolism and is considered essential for life. However, a number of desiccation‐tolerant organisms spread across the kingdoms of life challenge this thinking by surviving the loss of essentially all the hydrating water inside their cells. To perform this feat, desiccation‐tolerant organisms enter into a state of biostasis known as anhydrobiosis (from Greek for “life without water”) (Crowe & Clegg, 1973). In this anhydrobiotic state, organisms become ametabolic and remain so until rehydrated (Crowe & Clegg, 1973). Once rehydrated, even after years or in some cases decades, these anhydrobiotic organisms resume their normal metabolism and development (Crowe & Clegg, 1973).

Many anhydrobiotic organisms protect their cells from drying‐induced damage by accumulating non‐reducing sugars such as sucrose (Yathisha et al., 2020) and trehalose (Erkut et al., 2011; Laskowska & Kuczyńska‐Wiśnik, 2020; Mitsumasu et al., 2010; Tapia et al., 2015; Tapia & Koshland, 2014). The enrichment of disaccharides was long thought to be a universal feature of desiccation tolerance. However, several robustly anhydrobiotic organisms, such as tardigrades and rotifers, do not accumulate high levels, or in some cases any of these sugars during drying (Boothby et al., 2017; Hengherr et al., 2008; Lapinski & Tunnacliffe, 2003). Instead, these animals use a diverse array of intrinsically disordered proteins (IDPs) to provide adaptive protection against desiccation (Boothby et al., 2017; Denekamp et al., 2010; Piszkiewicz et al., 2019; Tripathi, 2012; Tripathi et al., 2012).

One example of desiccation‐related IDPs are Cytoplasmic Abundant Heat Soluble (CAHS) proteins (Boothby et al., 2017; Hesgrove & Boothby, 2020; Nguyen et al., 2022; Yamaguchi et al., 2012), which are employed by tardigrades to survive drying (Nguyen et al., 2022). Genes encoding CAHS proteins are induced in response to desiccation in tardigrade species that require preconditioning to survive drying (Boothby et al., 2017), or are constitutively expressed at high levels in tardigrade species that require little priming to tolerate water loss (Boothby et al., 2017; Yamaguchi et al., 2012). Here, we focus on CAHS D, a representative CAHS protein derived from the tardigrade *Hypsibius exemplaris*, which is required for anhydrobiosis in this animal (Boothby et al., 2017). CAHS D increases desiccation tolerance when heterologously expressed in yeast and bacteria, and is sufficient to protect sensitive biological material from desiccation‐induced damage in vitro (Boothby et al., 2017; Nguyen et al., 2022; Packebush et al., 2023; Piszkiewicz et al., 2019). CAHS proteins have been predicted to be disordered and this has further been demonstrated by NMR, hydrogen‐deuterium exchange experiments, and circular dichroism spectroscopy (Boothby et al., 2017; Eicher et al., 2022; Eicher et al., 2023; Malki et al., 2022; Wang et al., 2023; Yamaguchi et al., 2012).

Despite recent intense study of CAHS proteins, several important facets of their biology remain to be addressed or clarified.

As several groups have observed, some CAHS proteins, including CAHS D, can undergo a phase transition from the solution to solid gel state (Eicher et al., 2022; Eicher et al., 2023; Malki et al., 2022; Nguyen et al., 2022; Packebush et al., 2023; Sanchez‐Martinez et al., 2023; Tanaka et al., 2022; Yagi‐Utsumi et al., 2021). However, sequence features that underlie the gelation process have not been fully tested, and the proposed mechanisms that could underlie how CAHS proteins form fibrils and gels are in conflict. For example, Malki et al. (2022) suggested that the termini of CAHS D (CAHS 8 in their manuscript) lack secondary structure, while Eicher et al. (2022, 2023) and Wang et al. (2023) report that these regions contain beta‐structure which is speculated to be important for gelation. These disparate observations led the various groups to propose mechanisms of gelation for CAHS D that differ from one another (Eicher et al., 2022, 2023; Malki et al., 2022). These differences are seemingly accentuated when considering other CAHS proteins from additional tardigrade species (Tanaka et al., 2022; Yagi‐Utsumi et al., 2021).

Furthermore, the gelation of CAHS proteins has been speculated to play a role in the protective capacity of CAHS proteins during desiccation or osmotic stress, which Tanaka et al. (2022) propose could potentially be mediated through the formation of a cytoskeletal‐like network and stiffening of cells (Eicher et al., 2022; Malki et al., 2022; Tanaka et al., 2022; Wang et al., 2023; Yagi‐Utsumi et al., 2021). However, experiments perturbing the gelling capacity of CAHS D (or other CAHS proteins) and empirically linking this loss in gelation to a loss in protective capacity have not been conducted. Thus, to date any link between CAHS gelation and protective capacity is speculative as is any putative mechanism of protection.

Here we address the question of how CAHS D gels form as well as directly follow up on the work of others, especially the recent work of Tanaka et al. (2022) to ask if gelation is required for CAHS D mediated protection and if so by what mechanism(s)? To this end, we confirm that CAHS D forms concentration‐ and temperature‐dependent gels in vitro and that CAHS D forms gel‐like condensates in vivo during osmotic stress. CAHS D gelation restricts molecular motions of CAHS D itself as well as those of desiccation‐sensitive biological materials embedded within the gel. Using scanning electron microscopy (SEM) and small angle x‐ray scattering (SAXS), we characterize the structure of CAHS D hydrogels and find that they are composed of a reticular network of ~10 nm fibers. Using a combination of computational and biophysical approaches to characterize CAHS D, we show that this protein exists in a dumbbell‐like ensemble consisting of two collapsed terminal regions rich in transient β‐structure that are separated by a “Linker Region” that forms an amphipathic α‐helix. We find that while each region is necessary, none alone is sufficient to drive the solution‐to‐gel phase transition of CAHS D, suggesting that the protein's dumbbell‐like ensemble is essential for gelation. Using computational, biophysical, and molecular analysis, we arrive at a model of gelation where the amphipathic Linker Region drives bundling of CAHS D via helix–helix interactions, with higher‐order networks assembling via β–β interactions between the terminal regions. We find that upon osmotic stress CAHS D forms fibrillar condensates in vivo. Furthermore, CAHS D variants that perturb or modulate gelation in vitro also perturb or modulate fibrillization in vivo. During osmotic stress in cells, CAHS D fibrillization is required for resisting volume change, reducing metabolism, and improving survival. Interestingly, reduced metabolism during stress correlates strongly with increased survival rates, but reducing cell volume change does not. The condensation of CAHS D as well as its associated reduction of metabolism are reversible with the return to normal osmotic conditions. Thus, our data demonstrate that CAHS D gels form a novel cytoskeletal‐like assembly that promotes cellular survival during osmotic stress likely through the induction of reversible biostasis.

Our work advances our understanding of CAHS proteins' biology in two important ways. First, through a holistic combination of biophysical approaches and protein mutagenesis our work helps to resolve a conflict in the mechanism underlying CAHS D gelation, demonstrating empirically that emergent beta‐structure within termini promotes and is required for gelation. Second, our work advances our understanding of the mechanisms which may underlie CAHS D‐mediated protection of cells during osmotic shock. Our data confirms that CAHS proteins indeed assemble into a cytoskeletal‐like network that stiffens cells and reduces volume change during water loss, but we show that this does not correlate with increased survival. Rather, our data supports the notion that cytoskeletal‐like assembly is essential for reducing metabolism, a phenomenon which correlates strongly with cellular survival during osmotic stress and may be a robust mechanism for successful anhydrobiosis.

## 2 RESULTS

### 2.1 CAHS D forms a concentration dependent reversible gel

**FIGURE 1:**
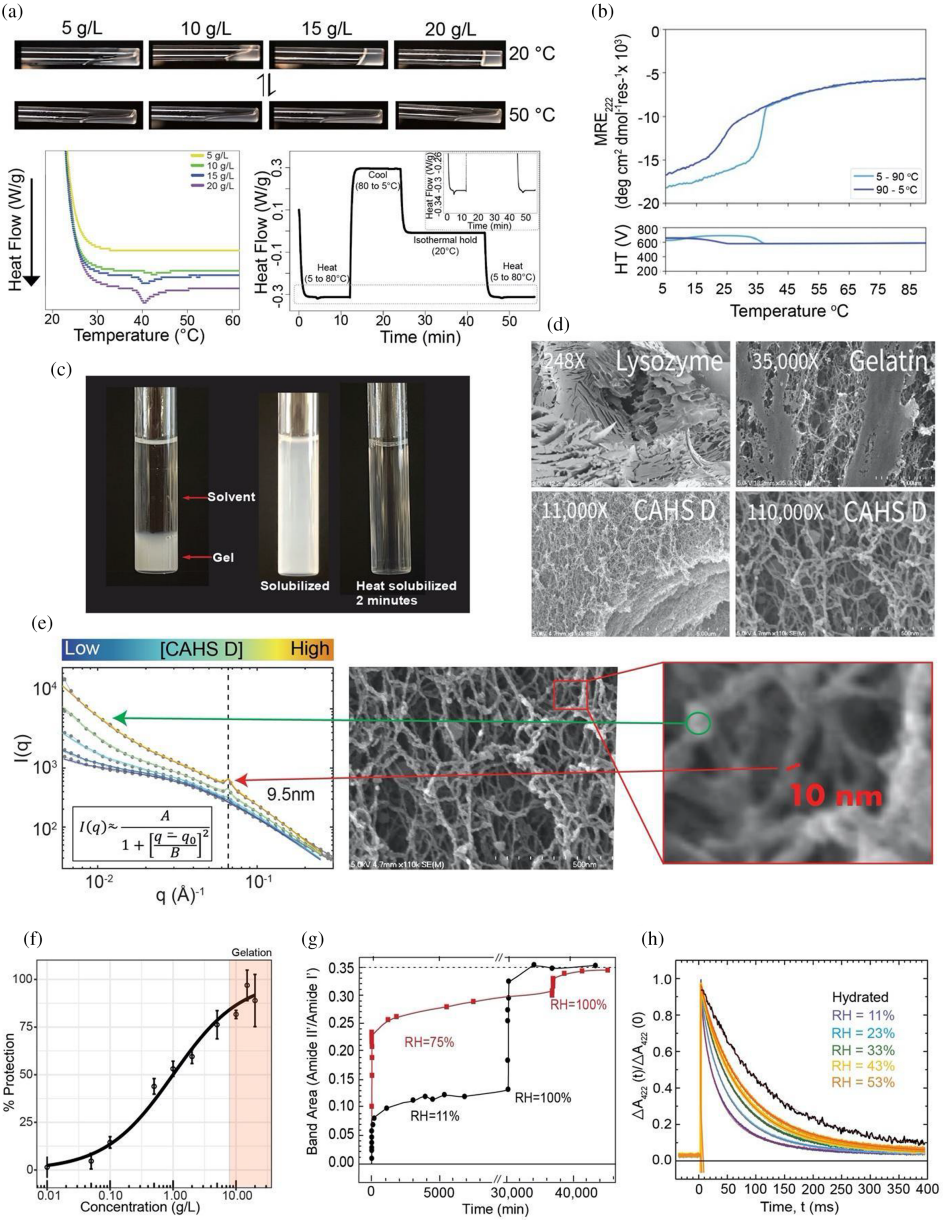
CAHS D forms a gel that immobilizes and stabilizes biological material in vitro. a) Concentration and temperature dependence of CAHS D gelation. CAHS D at 20°C (top panel) shows strong gelation at 20 g/L (0.8 mM), while at 50°C gelation is disrupted. Once cooled, the gel reforms (lower panel right). Differential scanning calorimetry shows a concentration‐dependent gel melt at ~40°C (lower left), which is reversible (lower right). (b) Thermal denaturation and cooling of CAHS D (0.7 mM, 17.7 g/L) shows a sharp and highly reversible change in mean residue ellipticity (MRE), indicating the dissociation (heating, light blue line) and reassociation (cooling, dark blue line) of the protein. (c) Dilution of 10 g/L (0.4 mM) CAHS D gel in 20 mM Tris buffer results in resolvation within ~2 min at 55°C. (d) SEM images of lysozyme, gelatin and CAHS D. CAHS D reticular gels are similar to the gel‐forming protein, gelatin, but distinct from that of the non‐gelling protein lysozyme. All SEM imaging was performed with proteins at 50 g/L. (e) Probing CAHS D gels at various concentrations with SAXS revealed an emergent structure of approximately 9.5 nm. The region of the SEM image magnified in red callout box shows a fiber of approximately 10 nm in width, corresponding well with the emergent peak found at increasing concentration in the SAXS plot (indicated by the red arrow and black dotted line). The green arrow highlights the change in the internal void space within a single fiber. See also Figure S1A,C. (f) Plot of lactate dehydrogenase (LDH) enzyme stabilization by CAHS D showing the percent protection of LDH by CAHS D as a function of concentration. *n* = 3, Error bars = standard deviation (adapted from Nguyen et al. 2022 (Nguyen et al., 2022)). (g) Kinetics of amide FTIR HDX in dried CAHS D matrices. Dried CAHS D matrices, equilibrated at RH = 6% were exposed to a D_2_O atmosphere at RH = 11% (black) or RH = 75% (red). Subsequently, both were transitioned to pure D_2_O atmosphere (RH = 100%), which demonstrated that both samples showed similar HDX when fully saturated. The dashed line represents the value of the amide II' band area normalized to the area of the amide I′ band of CAHS D in D_2_O solution. (h) Hydration‐dependent immobilization of a client protein within the CAHS D glassy matrix. Charge recombination kinetics after a light pulse (fired at *t* = 0) are shown for photosynthetic reaction centers in solution (black) and embedded into dried CAHS D gels held at varying humidity. Colored traces represent different relative humidity (purple, RH = 11%; blue 23%; green 33%; yellow, 43%; orange 53%). Continuous red curves represent best fit to Equation (1) (see Section 4).

CAHS D (also known as CAHS8_HYPEX; Uniprot: P0CU50) is a highly charged 227‐residue protein that is disordered (Boothby et al., 2017; Eicher et al., 2022; Malki et al., 2022; Wang et al., 2023) and is required for the tardigrade *H. exemplaris* to survive desiccation robustly (Boothby et al., 2017). Through the course of studying this protein, we and others have observed that CAHS D protein undergoes a solution‐to‐gel phase transition, which is in line with the behavior of other CAHS proteins (Figure 1a) (Eicher et al., 2022; Malki et al., 2022; Nguyen et al., 2022; Packebush et al., 2023; Sanchez‐Martinez et al., 2023; Tanaka et al., 2022; Yagi‐Utsumi et al., 2021).

To characterize the material properties of CAHS D further, we assessed the concentration dependence of gelation. In line with previous measurements (Eicher et al., 2022; Malki et al., 2022), using direct observation and differential scanning calorimetry (DSC), we found that CAHS D gelation is concentration dependent, with solutions below ~10 g/L (0.4 mM) remaining diffuse, increasing in viscosity between 10 and 15 g/L, and above ~15 g/L (0.6 mM), forming robust gels (Figure 1a). In addition to concentration dependence, gelation of CAHS D was observed to be reversible via heating and cooling (Figure 1a–c) as well as by dilution (Figure 1c). Reversibility suggests that gelation is driven by non‐covalent physical crosslinks, as opposed to covalent crosslinks (Almdal et al., 1993). The temperature dependence and reversibility of the solution‐to‐gel transition suggests that a favorable enthalpy change drives gelation, which is reinforced by the reversibility seen through re‐solvation (Figure 1c).

Next, we sought to better understand the morphology and structure of CAHS D gels using scanning electron microscopy (SEM) and small‐angle x‐ray scattering (SAXS). SEM analysis revealed that CAHS D gels form reticular networks of interconnected fibers (Figure 1d). This topology is similar to gels formed by gelatin, and distinct from solids formed by non‐gelling proteins, such as lysozyme (Figure 1d). SAXS analysis on the reticular mesh formed by CAHS D was performed using a concentration gradient of the protein (Figure 1e). As a function of increasing concentration, a scattering peak emerged and increased in intensity; namely, a well‐defined peak indicative of a repeating structure with a well‐defined length of ~9.5 nm (Figure 1e, red arrow). This feature size approximates the 12.3 (±2.3) nm fibers observed in our SEM imaging (Figure 1e, Figure S1A,B), considering that preparation of materials for SEM often adds ~2 nm of coating (Höflinger, 2013). These results support previous measurements made using atomic force microscopy (AFM) indicating that CAHS D fibers are ~10 nm in diameter (Malki et al., 2022). The large void regions between fibers are beyond the resolution of SAXS and manifest as a power law approaching small angles. Void spaces within the fibers appear as a mesh with a characteristic length scale (Figure 1e, Figure S1A), which shrinks in a concentration‐dependent manner from ~2.6 nm at low CAHS D concentration, to ~2 nm at higher concentrations (Figure S1C).

Taken together these data provide a detailed structural picture of CAHS D gels characterized by the non‐covalent polymerization of CAHS D into fibers, which is in line with preliminary reports (Eicher et al., 2022, 2023; Malki et al., 2022; Tanaka et al., 2022; Yagi‐Utsumi et al., 2021). From SAXS, SEM, and AFM (Malki et al., 2022) we infer that a single fiber has a well‐defined diameter of approximately ~9.5 nm, but internally has less well‐defined regions that lack density, the average size of which are ~2.3 nm.

### 2.2 CAHS D gels are protective and slow the motion of molecules in both the hydrated and dry state

We wondered whether the gelation of CAHS D correlates with its protective capacity during desiccation. Figure 1f shows the concentration‐dependent protection of the enzyme lactate dehydrogenase (LDH) (Boothby et al., 2017; Nguyen et al., 2022; Piszkiewicz et al., 2019) by CAHS D during desiccation (Figure 1f). Normally desiccation and rehydration results in a loss of enzymatic activity for LDH, but when coincubated with protective molecules, LDH protection can be preserved (Boothby et al., 2017; Nguyen et al., 2022; Piszkiewicz et al., 2019). While CAHS D is protective at concentrations below the point at which robust gelation is observed, optimal protection of LDH was only achieved with CAHS D at a sufficient concentration to form a continuous gel (Figure 1f), suggesting that gelation may promote robust protection during drying.

Given that some gels can vitrify, we wondered if the “vitrification hypothesis” could potentially help explain the protective function of CAHS D. The vitrification hypothesis, a cornerstone of the desiccation tolerance field, posits that as water is lost, protectants cause slowed molecular motion through the induction of a highly viscous state, leading to a protective, vitrified solid (Boothby et al., 2017; Buitink & Leprince, 2004; Crowe et al., 1998; Hesgrove & Boothby, 2020). This slowing down of molecular motion and function is thought to lead to a state of reversible biostasis (Boothby et al., 2017; Buitink & Leprince, 2004; Crowe et al., 1998; Hesgrove & Boothby, 2020).

To investigate the hindrance of molecular motions in CAHS D gels, we used Fourier‐transform infrared (FTIR) spectroscopy to perform hydrogen deuterium exchange (HDX) (Figure S1D). Hydration‐dependent HDX of the vitrified gel matrix was tested in dried and hydrated gels at low and high relative humidity (RH = 11% and RH = 75%, respectively; Figure 1g, Figure S1E). HDX experiments distinguish between a tightly packed, conformationally restricted matrix (slow exchange), and a loosely packed pliable matrix (rapid exchange) (Figure S1D). The kinetics of the initial major phase of exchange occurred much faster in high relative humidity (min) than low relative humidity (tens of min) (Figure 1g, Figure S1E). The limited total exchange observed at low relative humidity indicates tight packing of CAHS D within the matrix and strong inhibition of conformational fluctuations, which decreases accessibility of peptide amide groups to the deuterated atmosphere. The higher hydration level of the matrix at high relative humidity plasticizes the gel matrix, increasing conformational fluctuations of CAHS D leading to higher amide accessibility as shown by the high percent of deuterated amide groups. This is in line with our previous report that water acts as a plasticizer of vitrified CAHS D (Boothby, 2021). When both samples were kept at RH = 100%, they achieved the same level of HDX as a solubilized protein (Figure 1g, black dotted line), indicating that the total amides competent for exchange in each sample are equivalent when fully hydrated. These data further support the idea that molecular motion within CAHS D gels is restricted in a hydration‐ and concentration‐dependent fashion.

The observed restricted motion of the CAHS D itself when gelled led us to ask whether a desiccation‐sensitive macromolecule, such as a protein complex, could be immobilized and protected within a CAHS D gel during drying. To test this, we probed the conformational flexibility of a desiccation‐sensitive photosynthetic reaction center (RC) embedded in a CAHS D gel. The RC used (purified from the photosynthetic bacterium *Rhodobacter sphaeroides* (Feher et al., 1989)) is an ideal model system to probe conformational dynamics because when an RC is immobilized, the kinetics and reaction rate distribution of the charge recombination reaction are altered (Figure S1F) (Malferrari et al., 2015; McMahon et al., 1998; Palazzo et al., 2002). Conformationally restricted RCs not only have increased average rate constant (<*k>*), indicating a slowing of RC structural relaxation, but also increased width (*σ*) of the distribution of rate constants *k* (see Equation (1) in Section 4), further indicating RC immobilization over a large range of conformational substates (Malferrari et al., 2015; Palazzo et al., 2002). RCs embedded in vitrified CAHS D gels at RH = 11% showed increases in both <*k>* and *σ* (<*k>* = 35.5 s^−1^, *σ* = 23.8 s^−1^) compared to RC in solution (<*k>* = 8.8 s^−1^, *σ* = 3.6 s^−1^) (Figure 1h, Figure S1G,H). Increasing relative humidity in a stepwise fashion (RH = 23%–53%) showed resulting stepwise increases in RC conformational mobility (Figure 1h, Figure S1G,H). The results observed for the conformational dynamics of RCs incorporated into vitrified CAHS D matrices were similar to those obtained with RCs embedded into glassy matrices formed by trehalose (Figure S1G,H), a sugar known to function as an extraordinary bioprotectant via immobilization of sensitive biological material in a vitrified state (Malferrari et al., 2015; Malferrari, Savitsky, et al., 2016). Interestingly, CAHS D and trehalose glassy matrices incorporating RCs exhibit similar water contents (Figure S1I), in line with the report that CAHS D does not have any special water retentive properties (Sanchez‐Martinez et al., 2023).

Combined, these data demonstrate that optimal protection during drying is only achieved at concentrations where CAHS D forms a gel, and that gelation restricts the conformational flexibility of CAHS D itself as well as the molecular motions of larger desiccation‐sensitive macromolecules embedded within the gel‐matrix. These findings lead us to hypothesize that gelation may play a mechanistic role in tardigrade desiccation tolerance through the immobilization of sensitive biomolecules, which could induce biostasis.

### 2.3 Characterization of the CAHS D global structural ensemble

**FIGURE 2:**
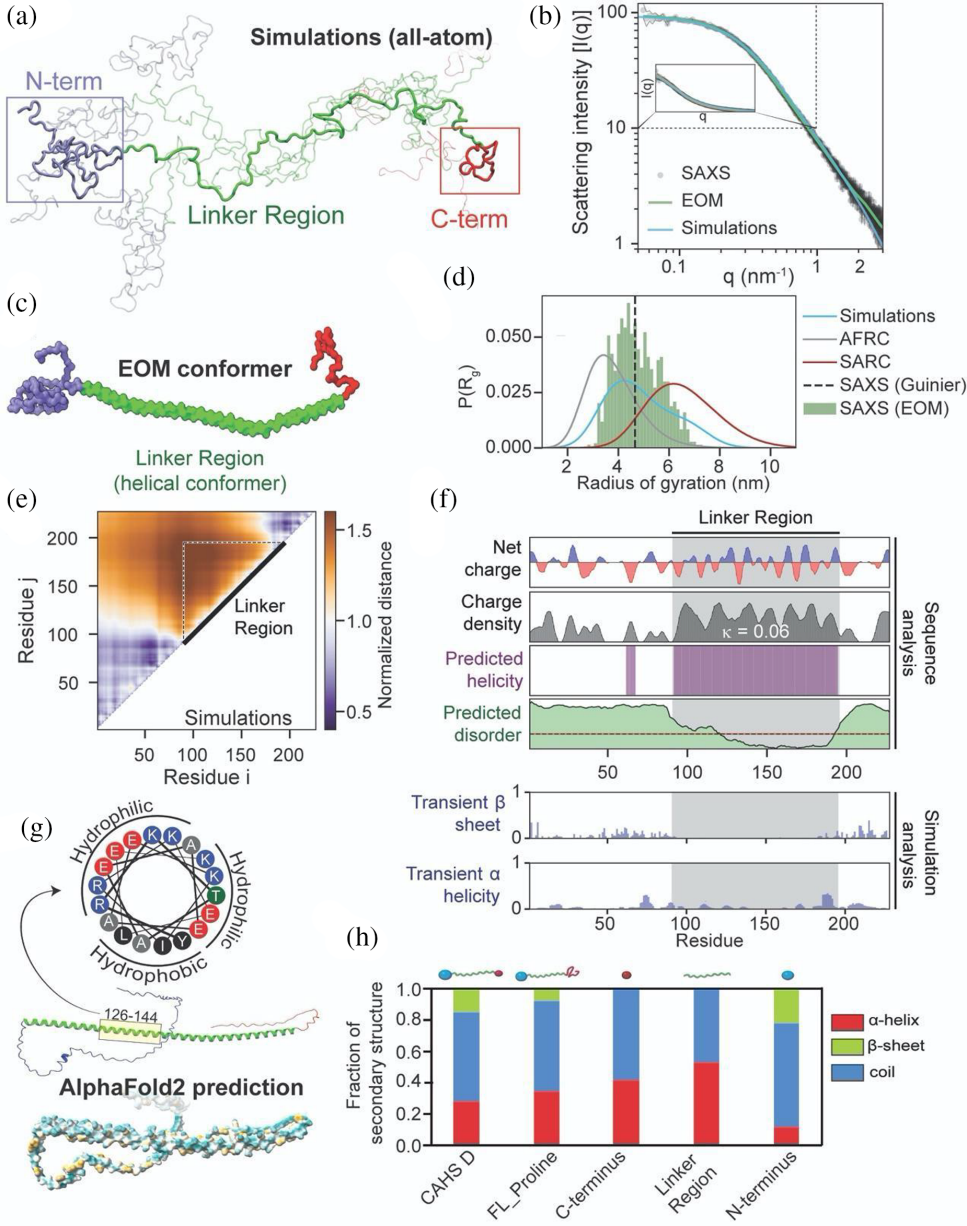
Global and local ensemble features of CAHS D. (a) Representative depiction of the CAHS D ensemble derived from all‐atom Monte Carlo simulation. Simulations predict two collapsed termini held apart by an extended linker. (b) SAXS data (black), EOM fitted scattering profile (green), and simulation‐derived scattering profile (blue) superimposed with one another. EOM and simulations are in good agreement with experimental data. See also Figure S2B–F. (c) Representative depiction of the CAHS D ensemble derived from EOM analysis. The three domains observed in simulations (a) are highlighted in blue (*N*‐terminus), green (linker), and red (*C*‐terminus). (d) Comparison of the radius of gyration distributions for monomeric CAHS D obtained from SAXS (dotted line), EOM (green bars), and Monte Carlo simulation (blue curve). For comparison, overlays for the Gaussian‐chain‐like AFRC model (gray) and self‐avoiding random coil (red) generated from sequence‐matched ensembles are shown. (e) A normalized distance map from CAHS D simulations compares the average inter‐residue distance with the expected inter‐residue distance if CAHS D behaved like a Gaussian chain. Hotter colors are further apart, cooler colors are closer. This analysis suggests that the terminal regions are largely collapsed, while the linker is expanded. (f) Localization of different sequence features within the CAHS D ensemble using bioinformatics and simulation analysis. (g) (bottom) Alphafold2 predictions of the CAHS D ensemble suggest high amphipathic helical propensity within the linker region (dark cyan most hydrophilic, dark golden most lipophilic). Helical wheel diagram of residues 126–144 of CAHS D (top). (h) Circular dichroism spectroscopy of CAHS D showing the fraction of different types of secondary structure for different variants.

To begin to empirically test mechanisms underlying CAHS D gel formation, which would allow us to experimentally probe the role of gelation in stress tolerance, we performed all‐atom Monte Carlo simulations of monomeric CAHS D. Previous reports have demonstrated that CAHS D is largely disordered in nature, but has a propensity to form transient secondary structure (Boothby et al., 2017; Eicher et al., 2022, 2023; Malki et al., 2022; Wang et al., 2023). Our simulations are consistent with a largely disordered protein, and suggest that CAHS D occupies a dumbbell‐like conformational ensemble, in which the *N‐* and *C‐*termini of the protein are relatively collapsed and are held apart by an extended linker region (Figure 2a).

To assess predictions of the global ensemble of CAHS D, we performed size exclusion chromatography‐coupled SAXS (SEC‐SAXS) of monomeric CAHS D. The linearity of the low‐q region after Guinier transformation supports the monodispersity of this sample (Figure S2A). In agreement with previous measurements (Malki et al., 2022), the ensemble radius of gyration from the Guinier approximation is 4.66 ± 0.07 nm. The scattering data were also analyzed using the Ensemble Optimization Method (EOM) to generate a SAXS‐derived distribution of radius of gyration (*R*_g_) values. From the EOM ensemble, the average *R*_g_ is 4.84 ± 0.92 nm, which is slightly larger than that estimated from the Guinier analysis (Figure 2b, Figure S2B–E). Consistent with these experimental results, the *R*_g_ predicted by ALBATROSS (a deep‐learning based sequence‐to‐ensemble predictor) is 4.75 nm (Lotthammer et al., 2023). Furthermore, EOM analysis suggests that CAHS D can sample dumbbell‐like conformations, in good agreement with the ensemble generated from Monte Carlo simulations (Figure 2c,d, Figure S2E).

Next, to improve the rigor of the CAHS D structural model, we refined the simulation ensemble using the experimental SAXS data. We employed a Bayesian/Maximum‐Entropy (BME) reweighting protocol in which the simulation frames are weighted such that the observables from the new simulation ensemble match those from the ensemble‐averaged SAXS data (Bottaro et al., 2020). Unless otherwise specified, all ensuing simulation analyses used the subsampled and reweighted ensemble (Figures S2F,G) (see Section 4 and Supporting Information).

The CAHS D ensemble dimensions are highly similar between simulations and experiments (Figure 2d). Notably, CAHS D is more compact than a self‐avoiding random coil (SARC R_g_, = 6.5 nm) and more expanded than a sequence‐specific Gaussian chain model (the analytical Flory random coil, AFRC) of the same length (AFRC *R*_g_ = 3.8 nm) (Figure 2d) (Alston et al., 2023). We compared the inter‐residue distances in the CAHS D simulations to those from the AFRC model and determined regions of local expansion and compaction. From the normalized distance map, the *N‐* and *C*‐termini are, on average, more compact than in the AFRC model, whereas the linker (residues 91–195) is more expanded (Figure 2e). Also considering the global CAHS D dimensions, these data implicate the Linker Region as the overall driver of ensemble expansion.

Analysis of the CAHS D sequence supports a linker charge‐driven model to spatially separate the *N‐* and *C*‐termini. The sequence parameter kappa (*κ*) ranges from zero to one and quantifies the charge patterning in a protein sequence, such that linearly well‐mixed sequences have low *κ* values, and sequences with segregated blocks of like charges have high *κ* values (Das & Pappu, 2013; Holehouse et al., 2017). Full‐length CAHS D is highly charged, and those charges are well‐mixed (*κ* = 0.086). However, the Linker Region has a lower *κ* of 0.06, making it more well‐mixed than over 99% of all tardigrade IDRs (Figure S2H,I). In other systems, highly charged well‐mixed IDRs (low *κ*) are more expanded than those within which charged residues are segregated (high *κ*) (Das & Pappu, 2013; Holehouse et al., 2017; Tedeschi et al., 2018).

By contrast, termini of CAHS D appear collapsed and compact relative to the linker in simulations (Figure 2a,e). Contact order analysis revealed that residues making inter‐amino acid contacts (contact order >0.02) were overwhelmingly hydrophobic (Figure S2J). Essentially all hydrophobic aliphatic residues (M, V, I, L) as well as a number of Q and N residues within the *N*‐ and *C*‐termini of CAHS D fall into this category and are likely to mediate the relative compactness of the terminal regions.

Taken together, our simulations, SAXS, and bioinformatics analyses suggest that CAHS D samples a dumbell‐like ensemble composed of two collapsed terminal regions bridged by a highly extended Linker Region, preventing intramolecular interaction between the *N‐* and *C*‐termini, and poising CAHS D for intermolecular interaction.

### 2.4 Characterization of the CAHS D local structural ensemble

While our simulations predict CAHS D is disordered, this does not preclude the existence of transient structure. Similar to Eicher et al. (2022, 2023) and Wang et al. (2023), our simulations suggest low levels of transient β‐strand in the *N*‐ and *C*‐terminal regions, while the Linker Region is predicted to contain transient α‐helix (Figure 2f, Figure S2K). Our simulations likely underestimate this helicity—bioinformatic predictions strongly indicate that the Linker Region is substantially helical, which is supported by recent solution‐phase NMR measurements that reveal a high degree of helicity within the linker region (Figure 2f, Figure S2K) (Malki et al., 2022). Furthermore, the Linker Region of CAHS D is predicted to be an amphipathic α‐helix with hydrophilic and hydrophobic faces (Figure 2g), as predicted for other CAHS proteins (Hesgrove & Boothby, 2020; Yamaguchi et al., 2012).

**FIGURE 3:**
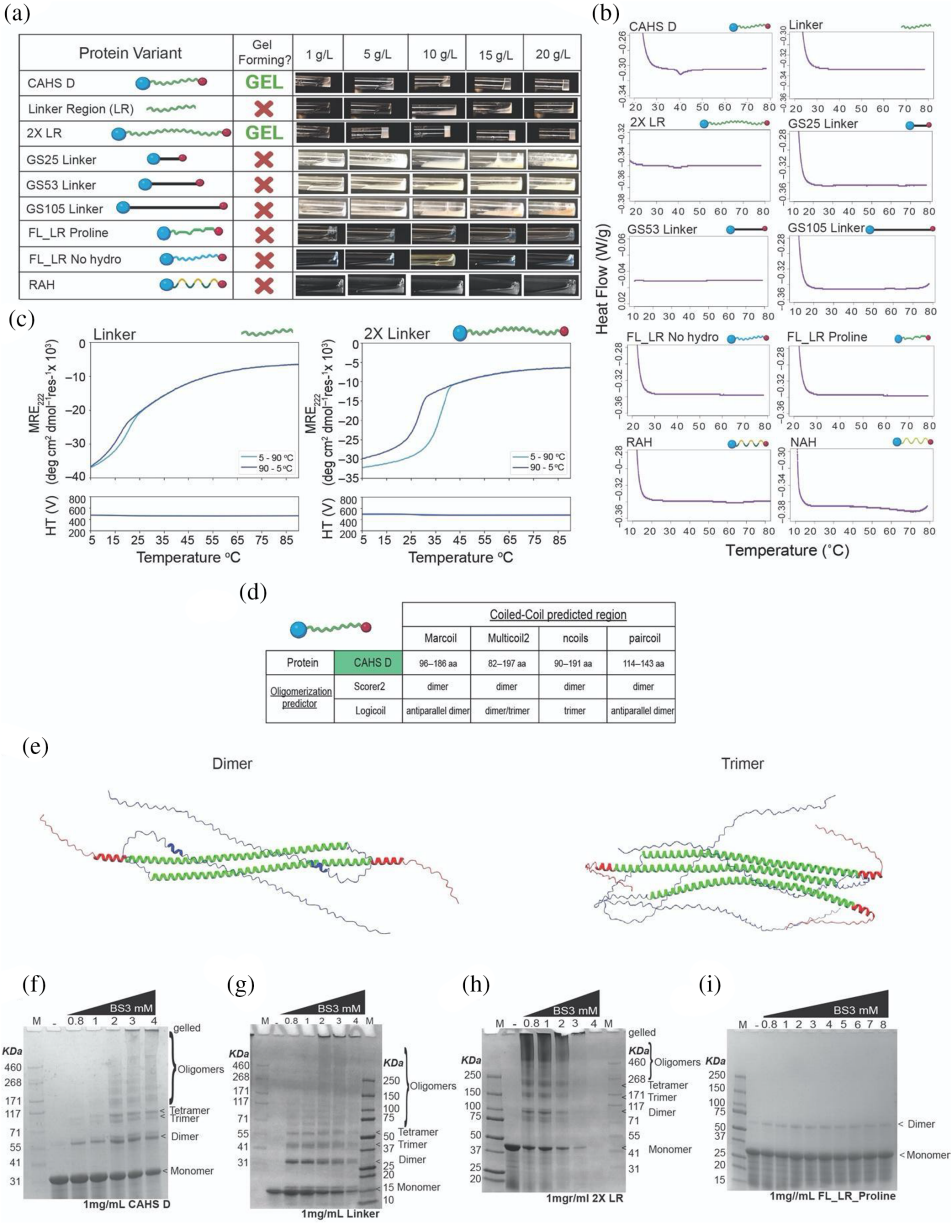
CAHS D's Linker Region is necessary but not sufficient for gel formation. (a) Table with graphical representations of CAHS D variants (column 1), whether or not they form a gel under the conditions tested (column 2), and images of solutions/gels formed by the variants at various concentrations (column 3). (b) Differential scanning calorimetry melt curves for CAHS D and its linker variants at 20 mg/mL. Only CAHS D and 2X Linker show melts characteristic of gels. (c) Thermal responses of the CD signals at 222 nm (ramping up, light blue line; and ramping down, dark blue line) for Linker Region (0.7 mM, 8.6 mg/mL) and 2X Linker Region (0.25 mM, 9.3 mg/mL) variants. Note that in both cases self‐association is observed, revealing that while the Linker Region alone can self‐associate it cannot form a gel. (d) Coiled‐coil predictions for CAHS D from a variety of predictors. (e) AlphaFold‐Multimer prediction of double and triple CAHS D complexes showing antiparallel arrangement of the molecules. (f) SDS‐page gel of 1 mg/mL CAHS D incubated with increasing amounts of BS3 amine ester crosslinker to visualize oligomeric state of CAHS D. (g) SDS‐page gel of 1 mg/mL Linker Region incubated with increasing amounts of BS3 amine ester crosslinker to visualize oligomeric state of Linker Region. (h) SDS‐page gel of 1 mg/mL 2X Linker incubated with increasing amounts of BS3 amine ester crosslinker to visualize oligomeric state of 2X Linker. (i) SDS‐page gel of 1 mg/mL FL_LR_Proline incubated with increasing amounts of BS3 amine ester crosslinker to visualize oligomeric state of FL_LR_Proline. In all crosslinking gels, the first lane has MW marker, the second lane has 1 mg/mL of proteins with no crosslinker showing monomeric state. Lanes 3 to end have 1 mg/mL of crosslinked proteins with increasing amounts of BS3 (0.8–to 4/8 mM) showing formation of dimers, trimers and high order oligomers.

We probed secondary structure experimentally in solution for each region of CAHS D using circular dichroism (CD) spectroscopy (Figure 2h, Figure S2L). CD spectra at non‐gelling concentrations confirm the largely disordered nature of full‐length CAHS D, with some propensity for α‐helix and β‐structure formation (Figure 2h). However, at concentrations when gelation is observed, CAHS D showed increased α‐helical character with the helix fraction ~44% at 700 μM (17.7 mg/mL) which is close to the fraction of 46% expected for CAHS D with a fully α‐helical Linker Region (Figure 1b, Figure S3E). Consistent with bioinformatic predictions, CD spectroscopy of truncation mutants of each CAHS D region confirmed substantial helical content in the Linker Region (~40%) (Figure 2h). Similarly to CAHS D, the Linker Region in isolation gave a strong α‐helical signal that increased to 89% helix at 700 μM (8.6 mg/mL) (Figure 3c, left panel, Figure S3F). Such high helix fractions for both CAHS D and the Linker Region at increased concentrations suggest that self‐assembly of CAHS D resulting in gelation is accompanied by the formation of helical bundles of the Linker Regions in agreement with previous works (Eicher et al., 2022; Malki et al., 2022; Tanaka et al., 2022). We note that the helicity of other CAHS proteins is also thought to be important for gelation (Tanaka et al., 2022; Yagi‐Utsumi et al., 2021).

**FIGURE 4:**
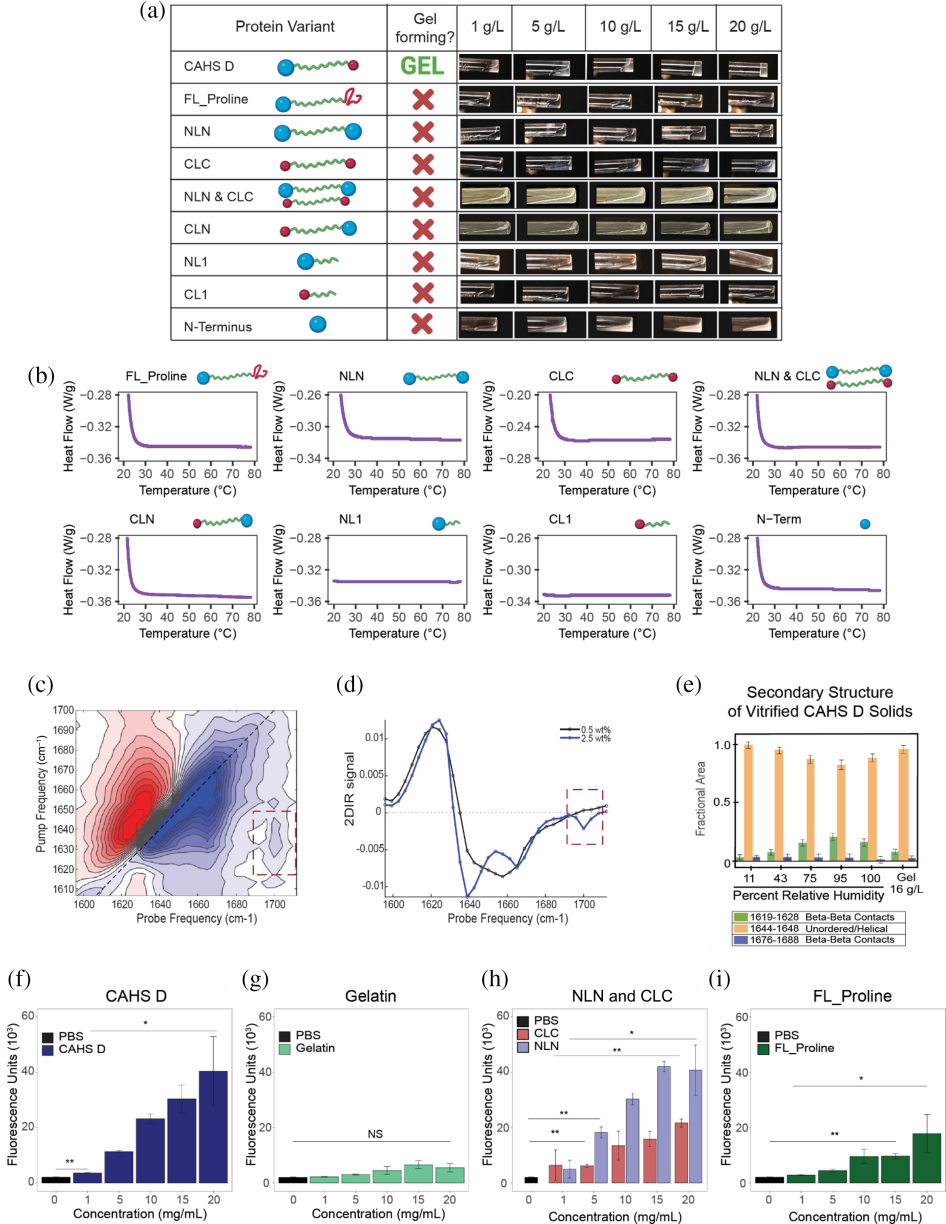
Heterotypic terminal domains are required for CAHS D gel formation. (a) Table with graphical representations of CAHS D variants (column 1), whether or not they form a gel under the conditions tested (column 2), and images of solutions/gels formed by the variants at various concentrations (column 3). (b) Differential scanning calorimetry melt curves for CAHS D and its terminal variants at 20 mg/mL. (c) 2D‐IR spectrum of CAHS D at a concentration above the gelation threshold (2.5 wt%). (d) horizontal slice through the 2D‐IR spectrum (obtained by averaging over the pump‐frequency range from 1625 to 1635 cm^−1^) of non‐gelled 0.5 wt% (black line) and 2.5 wt% (blue line) of CAHS D. Red dotted square shows the emergence of β‐sheet at gelation threshold 2.5 wt%. (e) Relative content in secondary structures of the CAHS protein as determined by FTIR analysis of the amide I′ band in a glassy matrix at different hydration levels (equilibrated at a relative humidity from 11% to 100%, as indicated) and in the hydrated gel state at concentrations of 16 g/L. The Gaussian sub‐bands centered in the wavenumber interval (1644–1648 cm^−1^) are attributed to unordered regions, while the Gaussian components peaking in the wavenumber intervals (1619–1628 cm^−1^) and (1676–1688 cm^−1^) are indicative of interprotein β‐sheet structures. (f) Thioflavin T (ThT) fluorescence as a function of concentration for CAHS D, (g) gelatin, (h) NLN, CLC and (i) FL_Proline. Error bars represent ±1 SD. Significance determined using a Welch's t‐test. All experiments presented used a minimum of three replicates. Asterisks represent significance relative to buffer controls and relative to 1 mg/mL of the proteins. *p < 0.05, **p < 0.01, ***p < 0.001, ****p < 0.0001, and NS is not significant.

Consistent with secondary structural prediction, CD spectroscopy indicates some β‐structure in the truncated *N*‐terminal Region (Figure 2h). Contrary to the secondary structure predictions, we did not observe residual β‐structure by CD spectroscopy in the isolated *C*‐terminal Region. However, we reasoned that the low β‐structure content observed by CD spectroscopy in the isolated *C*‐terminus could be caused by the loss of sequence context altering the conformational ensemble (Riback et al., 2018; Salvi et al., 2019; Theillet et al., 2014; Uversky, 2013). To test this possibility, we generated an additional CAHS D variant (FL_Proline). The FL_Proline mutant was designed to disrupt secondary structure in the *C‐*terminus via the insertion of three prolines into the three predicted β‐strands (Figure S2K) (Imai & Mitaku, 2005; Williams et al., 2004). The CD spectrum of FL_Proline showed >50% reduction in β‐structure content relative to wildtype (Figure 2h and Figure S2M), suggesting that the *C*‐terminus does have β‐structure when in the context of the full‐length CAHS D protein. Moreover, we found FL_Proline to be defective in gel formation (Figure S2N, Figure 4a,b). Eicher et al. (2022, 2023) and Wang et al. (2023) reported β‐structure in the termini of CAHS D while the report from Malki et al. (2022) suggests a lack of secondary structure in the termini of CAHS D. Our data are in line with the Eicher and Wang reports, suggesting that β‐structure exists within terminal regions of CAHS D. Reasons for these discrepancies may be due to differences in buffer conditions and/or protein purification methods.

Taken together, the results from CD spectroscopy support computational analysis and show helical content throughout CAHS D, with some localized, but likely transient, β‐structure content in the terminal regions. This finding helps to resolve a standing discrepancy in the field as to whether or not transient β‐structure is found within the terminal regions of CAHS D, which may be important during gelation.

### 2.5 The amphipathic nature of the Linker Region drives helical interactions and bundling of CAHS D

With an improved understanding of both the global and local ensemble properties of CAHS D, we set out to understand (i) which regions of CAHS D are necessary and sufficient for gel formation, and (ii) which secondary structural elements help mediate this process.

We began by testing the necessity and sufficiency of the Linker Region and its properties in mediating gelation, since several proposed mechanisms of CAHS D (as well as other CAHS proteins) gelation/fibrillization speculate that the linker, and in particular the helical nature of the linker, is involved in this process (Eicher et al., 2022; Malki et al., 2022; Tanaka et al., 2022; Yagi‐Utsumi et al., 2021). To this end, we generated a Linker Region (LR) variant lacking both the *N*‐ and *C*‐terminal regions (Figure 3a). This variant failed to form gels (Figure 3a,b), demonstrating that the Linker Region alone is not sufficient for gelation.

Next, we generated a variant we term 2X Linker Region (2X LR), which has the wildtype *N*‐ and *C*‐termini held apart by a tandem duplication of the wildtype Linker Region (Figure 3a). 2X LR gelled at a lower concentration than the wildtype CAHS D (Figure 3a,b), suggesting that while the Linker Region is not sufficient for gelation, it does play a role in the process.

Since gelation at a lower concentration with our 2X LR variant could be due to additional interactions between the extended Linker *or* to increased distance between terminal regions, we generated a series of glycine‐serine (GS) repeat variants. These GS variants replaced the endogenous Linker Region with GS repeats resulting in Linkers with predicted smaller (GS25; *R*_g_ = 4.04 nm), equivalent (GS53; *R*_g_ = 4.65 nm), or larger (GS105; *R*_g_ = 5.61 nm) *R*_g_ than the wildtype Linker Region (Figure 3a) (Moses et al., 2022; Sørensen & Kjaergaard, 2019). None of these variants gelled (Figure 3a,b) showing that sequence features and properties of the endogenous Linker Region, beyond merely holding terminal regions apart, are necessary for gelation.

To assess which sequence features of the Linker Region are essential for gelation, we began by testing the necessity of the linker's helicity in mediating gelation. Proline residues were inserted every 6–8 amino acids within the Linker Region to break the helicity of the full‐length CAHS D (FL_LR Proline; Figure 3a). As expected, we observed that these mutations led to a decrease in helicity (Figure S3A) and our FL_LR Proline variant failed to gel (Figure 3a,b), again indicating helicity of the Linker Region is important for this process.

We probed the characteristics of the wildtype Linker Region helix that are important for gelation further by assessing the sequence chemistry of the Linker Region in mediating this process. To this end, we generated a variant consisting of the full‐length protein with all the hydrophobic residues in its Linker Region substituted for glycine residues (FL_LR No hydro), which also failed to gel (Figure 3a,b, Figure S3B). Next, we generated two variants that maintained the hydrophobicity of the Linker Region, while shuffling the distribution of hydrophobic and hydrophilic residues resulting in a non‐amphipathic helix (NAH) or a robust amphipathic helix (RAH), both of which also failed to gel (Figure 3a,b, Figure S3C,D). We note that the NAH variant expressed and purified with low yield, leading us to characterize its gelation only by DSC, due to the large input of material required for direct observation as presented in Figure 3a.

Taken together, these results demonstrate that while the Linker Region is not sufficient for gelation, it is necessary, and furthermore that the predicted amphipathic helix within this region is highly tuned to mediate this phenomenon.

It has been speculated that the Linker Regions of different CAHS D proteins could interact with one another to help mediate fiber formation and gelation (Eicher et al., 2022; Malki et al., 2022). As mentioned above, the high helix fractions for both CAHS D (44%) and LR (89%) at increased concentrations suggest that self‐assembly of CAHS D resulting in gelation might be accompanied by the formation of helical bundles of the Linker Region. To test this, we performed CD spectroscopy on CAHS D and both the LR and 2X LR variants at concentrations at which wildtype CAHS D forms robust gels in controlled heating and cooling experiments (Figures 1b and 3c). All three proteins showed cooperative unfolding upon heating indicative of self‐assembly (Figures 1b and 3c). Both processes were highly reversible (Figure S3E–G). Interestingly, the midpoint melting temperature (T_M_) of 2X LR variant was similar to that for CAHS D (36–37°C); that is, linker length does not affect the melting point (Figures 1b and 3c). The LR alone at high concentrations showed a similar cooperative transition, but with a significantly lower T_M_ of 20°C. This suggests that while the linker alone can undergo self‐assembly (but not gelation) there is a significant role of the terminal domains in the self‐assembly and gelation of CAHS D and 2X LR.

Next, we wondered what the nature of the Linker‐Linker interaction might be. Previous reports have suggested that coiled‐coil interactions might drive CAHS protein gelation (Eicher et al., 2022; Malki et al., 2022; Tanaka et al., 2022; Yagi‐Utsumi et al., 2021). Since CAHS proteins possess amphipathic helices with strong hydrophilic and hydrophobic faces (Figure 2g) (Hesgrove & Boothby, 2020), and since the burial of hydrophobic residues is known to be a strong driver of α‐helical coiled‐coil formation (Monera et al., 1996; Woolfson & Alber, 1995), we assessed the predicted ability of CAHS D to form coiled‐coils using four different prediction tools for coiled‐coil propensity and oligomerization (Figure 3d, Figure S3H). CAHS D is predicted to form coiled‐coil dimers and trimers, with antiparallel dimers being predicted by most tools (Figure 3d). Furthermore, AlphaFold‐Multimer predictions with two or three copies of the CAHS D sequence show antiparallel helical interactions between Linker Regions (Figure 3e).

While predictive tools largely suggest an antiparallel dimeric assembly of CAHS D, we directly assessed the oligomerization state of CAHS D using an amine ester crosslinker (Figure 3f). SDS‐PAGE denaturing gels show a formation of dimers at low crosslinker concentration and higher order oligomers (including faint bands indicating trimerization) upon an increase of crosslinker concentration.

We also assessed the oligomerization state of the Linker Region (Figure 3g), 2X LR (Figure 3h) and FL_LR_Proline (Figure 3i) by amine ester crosslinking. The Linker Region shows formation of dimers, trimers and tetramers at low concentration and higher order oligomers upon increasing crosslinker concentration. This means the Linker Region by itself is enough to drive helix–helix interactions, but not gelation. 2X LR shows even at low crosslinker concentration of 0.8 mM, dimers and higher‐order oligomers, in line with the capacity of this protein to oligomerize and form gels at low concentrations. FL_LR_Proline variant shows very faint dimer bands.

Combining data from the experiments described above, we conclude that the Linker Region of CAHS D is necessary but not sufficient for its gelation and that gelation, at least in part, relies on the helicity and amphipathic nature of the linker to form antiparallel dimers.

### 2.6 β‐Structure interactions between CAHS D termini drive gel‐network formation

Since the Linker Region of CAHS D is necessary but not sufficient for gelation, we wondered which other region(s) of the protein are required to drive gel formation.

To test the necessity of the terminal regions of CAHS D in mediating gelation, we produced variants that have either deleted or swapped *N*‐ and/or *C*‐terminal Regions (Figure 4a). We observed that removal of either the *N*‐ or the *C*‐terminal Region (NL1 and CL1), or disruption of the *C‐*terminus by inserting proline residues (FL_Proline variant) resulted in a failure to gel (Figure 4a,b, Figures S2N and S4A). Likewise, replacing the *N‐*terminal Region with the *C*‐terminal Region (or vice versa, NLN and CLC variants) resulted in a loss of gelation (Figure 4a,b, Figure S4A), implying that heterotypic interactions between the two regions are likely essential for gelation, in line with our observation that helical interactions within the Linker Region are predicted for CAHS D to take place in an antiparallel fashion (Figure 3d,e).

Since gelation appears to require heterotypic termini (e.g., an *N*‐ and a *C*‐terminus), we mixed our NLN and CLC variants in a 1:1 molar ratio to test if just the presence of both termini will induce gelation. Mixing of NLN and CLC also resulted in no gelation, implying that the *N*‐ and *C*‐termini likely need to be in context with the linker sequence and that antiparalel dimerization of the Linker region is also likely needed.

To get more insights into the nature of the heterotypic interactions between the termini, we made a mutant swapping the order of the termini, generating a variant we term CLN. The CLN mutant has endogenous sequence of the *C*‐terminus at the —NH_2_ end of the protein, and conversely has the endogenous *N*‐terminal sequence at the —COOH end of the protein. CLN also failed to gel (Figure 4a,b, Figure S4A), implying that the context of the termini with respect to the linker is important.

To determine what ensemble structure within the termini drives gel formation, we performed femtosecond two‐dimensional infrared (2D‐IR) spectroscopy on CAHS D solutions at concentrations below and above the critical concentration for gelation. 2D‐IR cross peaks are reliable markers for the presence of β‐structure (Cheatum et al., 2004; Demirdöven et al., 2004), especially in the presence of other secondary structures in the protein (Giubertoni et al., 2022). Our analysis shows the emergence of β‐structure in gelled, but not in non‐gelled CAHS D (Figure 4c,d).

Next, we assessed how the conformation of CAHS D changes going from the gelled to the desiccated state using FTIR. We observed that β‐structure increased in the hydrated state as a function of concentration (Figure 4d). This increase continued in the drying hydrogel (95%–100% RH) but decreased at lower hydration levels (11%–75% RH) (Figure 4e). This implies that there is an optimal hydration level for stabilizing β‐structured contacts, which may relate to the need for higher stability while the matrix is undergoing the final stages of drying or the early stages of rehydration.

Finally, to determine whether higher β–β interactions correspond with the expected β‐structure content of the termini, and the ability of these to interact, we assayed CAHS D at different concentrations with thioflavin T (ThT). ThT is used as a fluorescent indicator of amyloid fibrils (Biancalana et al., 2009; Wu et al., 2009), but more generally reports on β‐structured assemblies (Kar et al., 2021; Namioka et al., 2020; Peccati et al., 2017). We observed steady increases in ThT fluorescence intensity as a function of CAHS D concentration (Figure 4f), consistent with higher β‐structure as gelation progresses. ThT signal in samples containing gelatin, which forms gels exclusively via entwined α‐helices without the need for β–β contacts (Sajkiewicz & Kołbuk, 2014), was not significantly different from buffer controls (Figure 4g).

To determine how each terminus contributes to β–β interactions, we probed three variants using the ThT assay; NLN and CLC, which have two homotypic termini, and FL_Proline, which has disrupted *C*‐terminal β‐structure (Figure 4a, Figure 2h, Figure S2M). ThT labeling of NLN and CLC variants showed a concentration‐dependent increase in fluorescence, with higher fluorescence observed in NLN than CLC (Figure 4h), which is consistent with the predicted and measured degree of β structure in each terminus (*N* vs. *C*) (Figure 2h). The ThT fluorescence of FL_Proline was less than variants with two endogenous termini but more than the gelatin control, as would be expected of a protein that is only competent to form β–β interactions with one terminus (Figure 4i). It is important to note that ThT fluorescence does not report on the strength of interactions, simply the relative quantity of interactions. Therefore, the pattern observed for ThT fluorescence; NLN > CAHS D > CLC > FL_Proline, follows the predicted β‐structure content of these variants (Figure 2h). Combined with our determination that NLN and CLC variants do not gel, these data suggest that while N–N and C–C interactions can occur, they are not able to induce gelation as is observed with N–C interactions, more over the context of the sequence between the *N*‐terminus and the beginning of Linker region and the end of Linker region and beginning of *C*‐terminus seem to be important since our CLN variant and mixtures of NLN and CLC variants fail to gel.

Taken together, these data empirically demonstrate that heterotypic interactions between β‐structure in the *N*‐ and *C*‐terminal Regions are necessary for the gel‐forming abilities of CAHS D. However, neither the *N‐* or *C‐*termini are sufficient themselves for gelation (Figure 4a,b, Figure S4).

### 2.7 Gelation of CAHS D occurs through helical interactions of the linker to form bundles which polymerize via β–β interactions

Based on the experiments and data described above, we conclude that both the interactions between Linker Regions and β–β interactions between heterotypic terminal regions of CAHS D are necessary to form dimers that ultimately assemble into higher‐order structures, that is, fibers with a diameter of ~9.5 nm.

**FIGURE 5:**
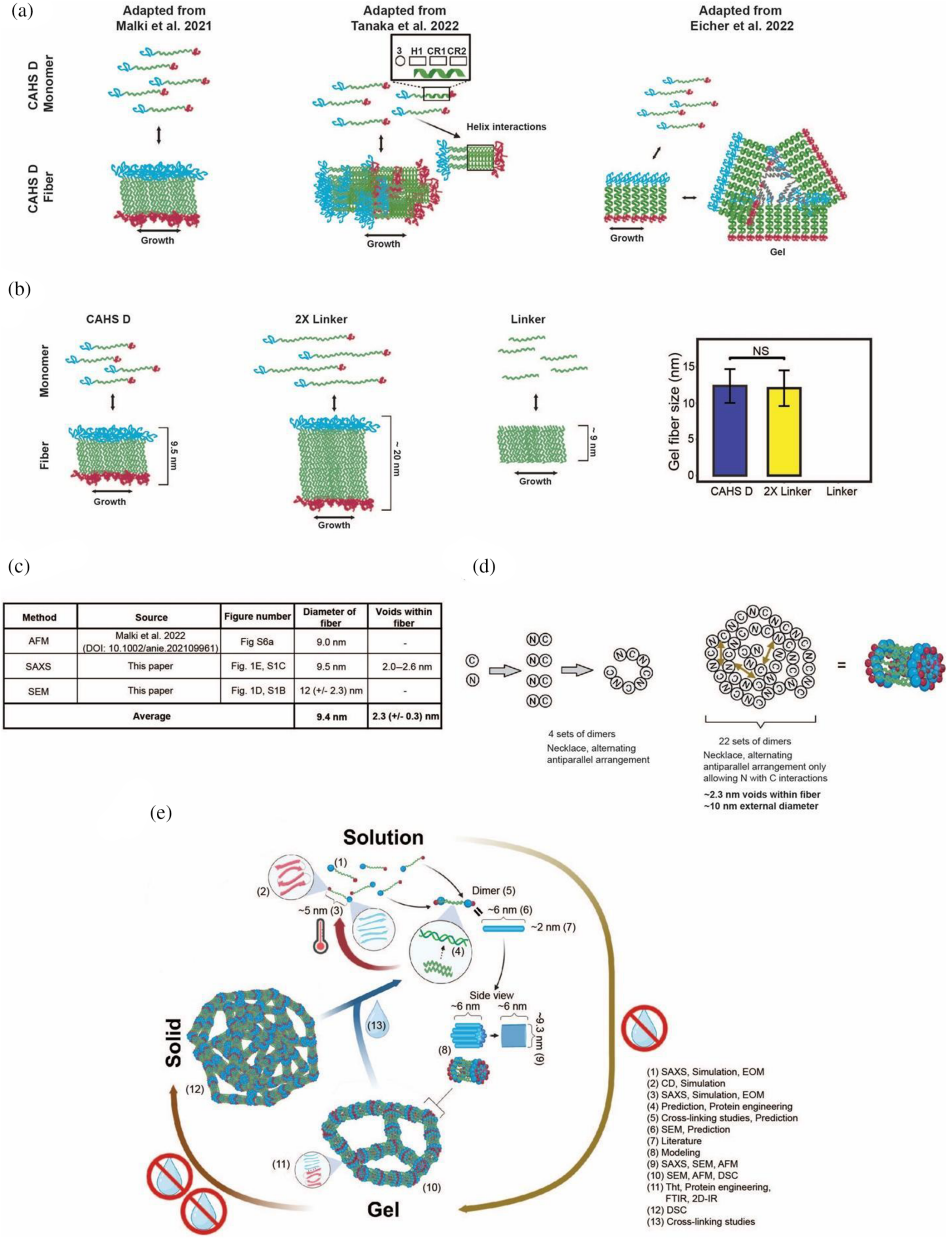
Model of in vitro CAHS D gel formation. (a) Schematic representation of leading hypotheses on how CAHS gel formation could be mediated. (b) Schematic representation of expected gel fiber diameters given the helical stacking hypothesis (left). Quantification of fiber diameters from CAHS D and 2X Linker gels (right). (c) Table summarizing empirical measurements of CAHS D gel fiber diameter. (d) Modeling of a necklace arrangement of CAHS D monomers and dimers. (e) Working model of CAHS D dimerization and gel formation. Numerical annotations note where evidence for model elements come from.

It has been proposed that fibrillization of CAHS proteins might be mediated exclusively by the stacking of helices (Malki et al., 2022) or coiled‐coil interactions between the *C*‐terminal ends of these helices (Tanaka et al., 2022) (Figure 5a). Our empirical demonstration that CAHS D gelation requires both its *N*‐ and *C*‐terminal regions, and that β‐structure within these regions is necessary for this, rules out these possibilities as the exclusive mechanisms mediating CAHS D gelation. Nevertheless, we probed the mechanism of fiber formation further by examining the morphology and dimensions of gels formed by our 2X Linker variant (Figure S5A).

In the model put forward by Malki et al. (2022) one would expect that the width of gel fibers should be approximately doubled in our 2X Linker variant as compared to the wildtype variant (Figure 5b). However, quantitative analysis of scanning electron micrographs of fibers formed by wildtype CAHS D and the 2X Linker variant showed no statistical difference in gel‐fiber width (12.3 ± 2.3 vs. 12.1 ± 2.4 nm, respectively) (Figure 5b, Figure S5A). One would also expect that if the termini do not play a role in gelation as stated in the helical stacking hypothesis, our Linker Region, FL Proline, NLN, and CLC variants would also form fibers (Figure 5b), but they do not (Figure 3a,b and Figure 4a,b). Additionally, simulation, SAXS, and EOM indicate that the *R*_g_ of monomeric CAHS D is ~5 nm (Figure 2d), well short of the ~9.5 nm fiber diameter as measured by SAXS (Figure 1e), SEM (Figure S1B), and AFM (Malki et al., 2022). This logic would suggest that the hypothesis posed by Malki et al. (2022) is unlikely, since it does not fit with the requirement for the terminal regions, nor is it congruent with empirically determined dimensions of CAHS D gel fibers. Similarly, a mechanism by which CAHS D gels exclusively through helical interactions in the *C*‐terminus, such as that proposed by Tanaka et al. (2022) can be ruled out due to the necessity of having both the *N*‐ and *C*‐terminal regions of the protein as well as the necessity of β‐structured interactions between these regions (Figure 4a–i). It should be noted that at the time of their publication, both the Malki et al. (2022) and Tanaka et al. (2022) hypotheses were consistent with the available data. It should also be noted that Tanaka et al. (2022) examined the gelation of a different CAHS protein (CAHS 3 from R. varieornatus), which is distinct from CAHS D.

Eicher et al. (2022) provide a model of CAHS D fiber formation that requires the helical association of Linker Regions, while networking of these fibers into a gel requires β–β interactions (Figure 5a). Based on *R*_g_ measurements of CAHS D as well as SEM and SAXS measurements presented here and previous AFM (Malki et al., 2022) characterization of CAHS D fibers, this model of fiber formation is lacking, since CAHS D has an *R*_g_ (~5 nm) of about half the width of a CAHS D fiber (~10 nm).

Since none of the previously proposed hypotheses of CAHS D fiber formation/gelation fulfill the requirements provided from empirical evidence, we sought to develop a model which would. Combining results from global and local ensemble studies, protein engineering, imaging, material and biophysical characterization, we arrive at a model of CAHS D gelation that is consistent with empirical measurements of CAHS D fiber measurements (Figure 5c–e). We proposed that in solution monomeric CAHS D forms antiparallel, staggered, dimers with a length of ~6 nm (Figure S5B) and other higher‐order structures in a concentration‐dependent fashion through the association of linker regions of adjacent CAHS D proteins (Figure 3d, f and Figure S3H). Considering the ~9.4 nm diameter and ~2.3 nm voids of gel fibers (Figure 1e, Figure S1C), we invoke a necklace model (Figure 5d) in which 22 dimers associate and supercoil into a bundle with these dimensions. Twenty‐two dimers is consistent with the number of dimers (assuming a coiled‐coil diameter of ~2 nm) required to obtain a supercoiled bundle with a diameter of ~9.4 nm. Bundles of antiparallel dimers then polymerize through end‐to‐end β–β interactions to form a gel (Figure 5e). Further desiccation of the gel results in solidification into a non‐crystalline amorphous solid (Boothby, 2021), which is expected as CAHS gels themselves, like many other gels, are amorphous (Douglas, 2018; Horkay et al., 2019). Finally, rehydration results in a rapid re‐solvation and dissipation of the gel as CAHS D molecules rapidly go back into solution (Figure 5e, Figure S5C,D).

**FIGURE 6:**
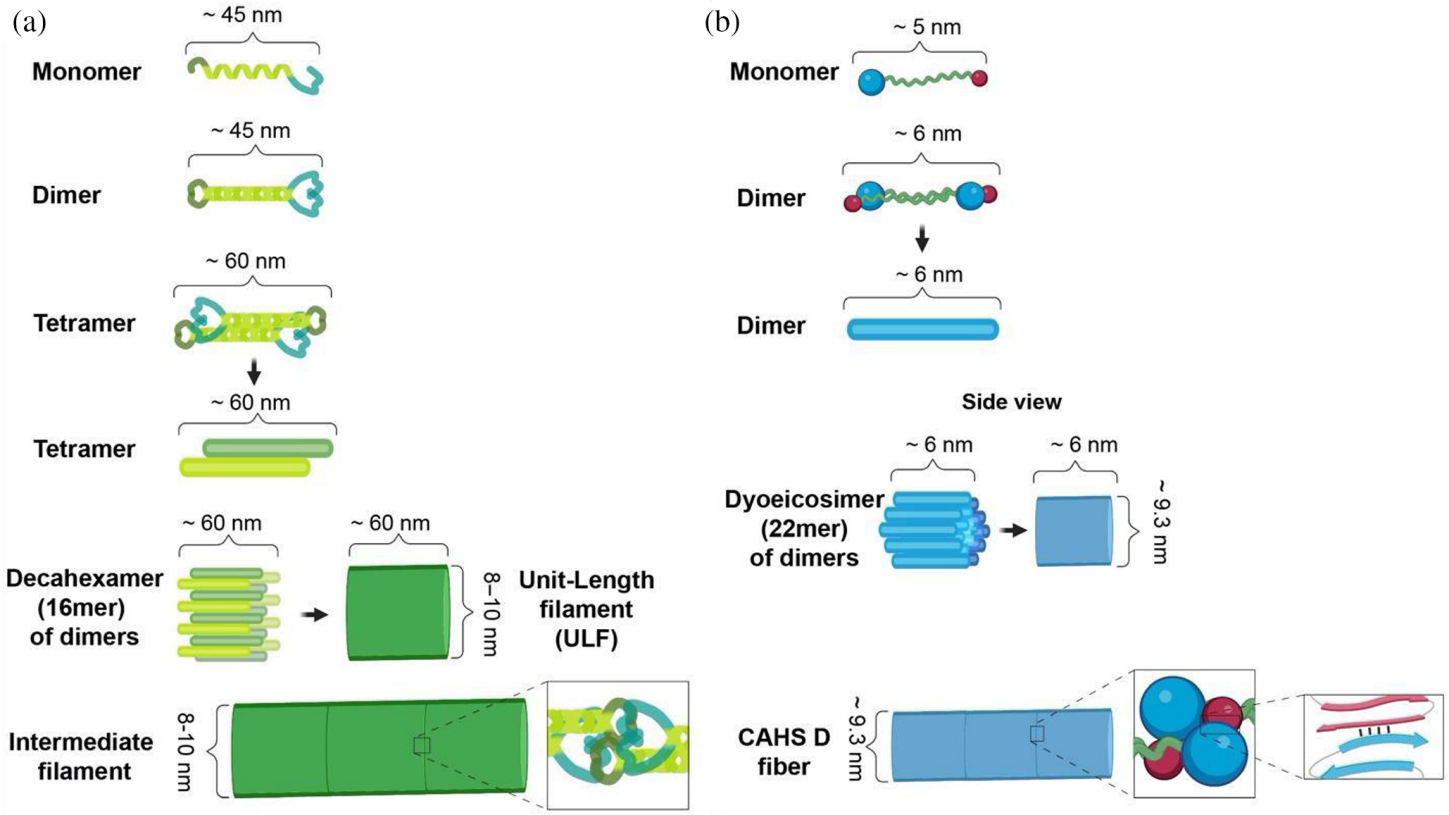
Comparisons of proposed mechanisms of intermediate filament and CAHS D fiber formation. (a) Schematic representation of intermediate filament formation (adapted from (Qin et al., 2009)). (b) Schematic representation of proposed mechanism of CAHS D fiber formation.

Previous commentary has highlighted the similarities between CAHS fibers and intermediate filaments (Figure 6a) (Tanaka et al., 2022). Indeed, our proposed mechanism of CAHS D fiber formation shares striking similarities with the mechanism of intermediate filament formation (Figure 6a,b). This reinforces the intriguing possibility brought up by Tanaka et al. (2022) that CAHS gel formation may serve to promote the assembly of a stress‐induced cytoskeletal‐like network in cells (Tanaka et al., 2022).

### 2.8 CAHS D forms gel‐like condensates in living cells during osmotic stress

To begin to directly address the question brought up by Tanaka et al. (2022) of whether there are functional similarities between CAHS proteins and intermediate filaments, we sought to understand whether or not CAHS D gels in vivo. CAHS proteins from a different tardigrade species have been reported to form condensates in cells upon osmotic shock, improve survival of osmotically shocked cells, and osmotically shocked cells expressing these proteins stiffen (Tanaka et al., 2022; Yagi‐Utsumi et al., 2021). However, the sequence features underlying this condensation have not been fully elucidated nor has the role of condensation in cellular stress tolerance or stiffening been tested empirically.

To assess if CAHS D can also form condensates in cells, and if so, to understand the sequence features that drive this behavior, we generated a stable human embryonic kidney (HEK) cell line expressing an mVenus:CAHS D fusion protein. We also generated a stable cell line expressing mVenus alone as control.

**FIGURE 7:**
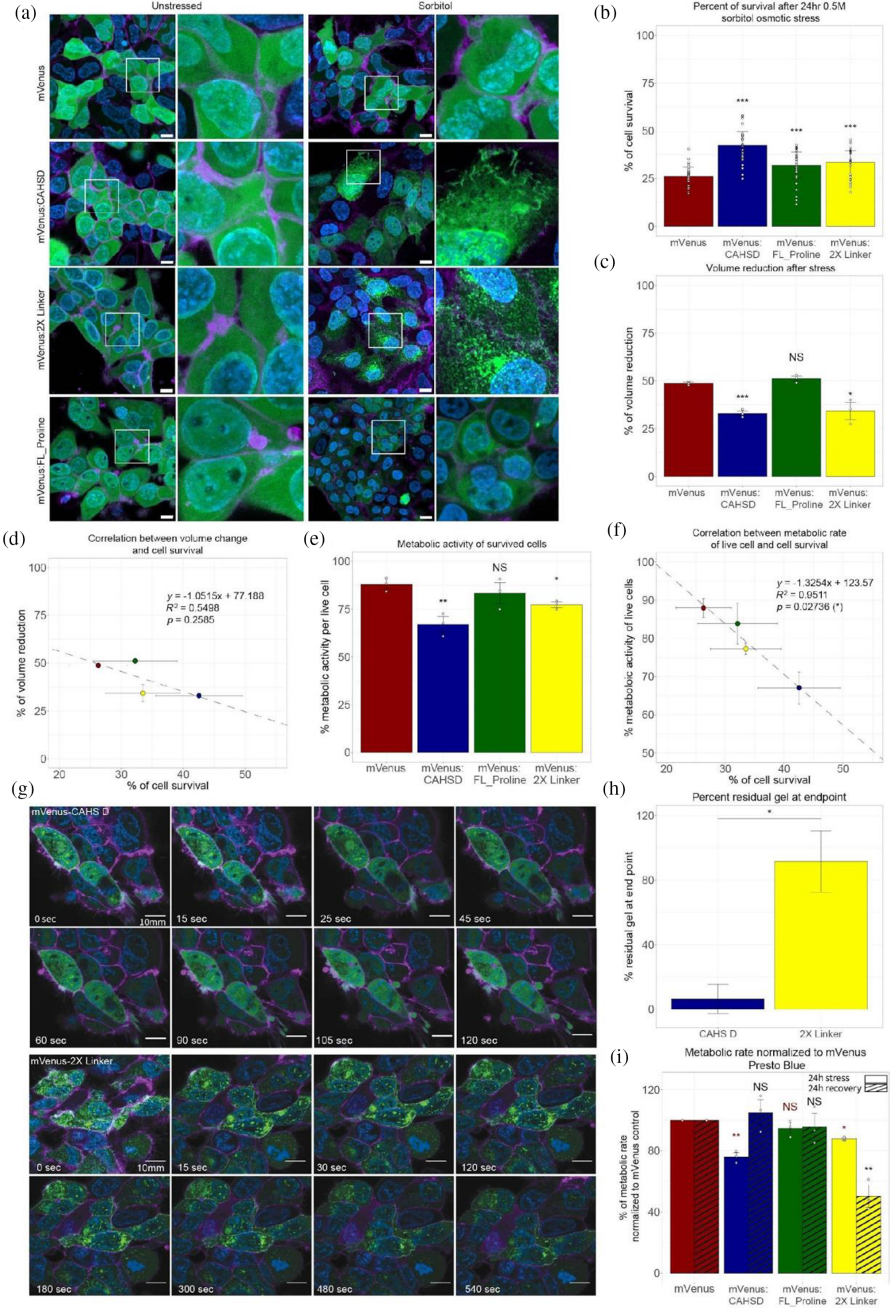
Condensation of CAHS D induces reversible biostasis and increases survival during osmotic shock. (a) Fluorescence microscopy of human embryonic kidney cell lines expressing mVenus or mVenus fusions to CAHS D variants. The left two columns show cells and insets of cells imaged under non‐stressed conditions. The right two columns show cells and insets of cells treated with 0.5 M sorbitol. Green = mVenus, blue = DNA, purple = cell membrane. Scale bar = 10 μm. (b) Combined survival data from Calcein AM/Propidium iodide and Hoechst/Propidium iodide survival assays (data from each individual assay shown in Figure S6C,D). (c) Cell volume reduction during osmotic stress. (d) Correlation between cell volume change and survival during osmotic stress. (e) Quantification of metabolic activity of living cells during osmotic stress using Presto Blue HS assay (for XTT metabolic assay data see Figure S6I,J). (f) Correlation between reduction in metabolic activity and survival during osmotic stress. (g) Confocal time‐lapse imaging of CAHS D and 2X Linker condensates in cells recovering from osmotic stress. Scale bar = 10 μm. (h) Quantification of residual condensational at end‐point of time‐lapse experiment (120 s for CAHS D and 540 s for 2X Linker). (i) Normalized metabolic rates of alive cells to mVenus control cells during osmotic stress (solid bars) and after 24 h recovery (striped bars) (For XTT assay data see Figure S6J). Error bars represent average deviation. Significance determined using a paired T. Test, asterisks represent significance relative to mVenus control cells. *p < 0.05, **p < 0.01, ***p < 0.005, and NS is not significant. In figure (i) red color statistics represent significance relative to mVenus stressed cells, and black color statistics represent significance to mVenus recovered cells).

Both cell lines expressing mVenus alone and our mVenus:CAHS D fusion show diffuse distributions of protein in the cytoplasm of non‐stressed HEK cells (Figure 7a). However, while the localization of mVenus remained diffuse within the cytoplasm of osmotically shocked HEK cells, mVenus:CAHS D localized into fiber‐like condensates (Figure 7a).

Next, we assessed if sequence features that promote gelation of CAHS D in vitro also drive condensation in vivo. For this, we established stable HEK cell lines expressing fusions of mVenus and the FL_Proline and 2X Linker variants and observed their subcellular localization before and during osmotic stress. Both mVenus:FL_Proline and mVenus:2X Linker were diffusely distributed through the cytoplasm, similar to mVenus and mVenus:CAHS D, in unstressed cells (Figure 7a, Figure S6A,B). However, upon osmotic shock mVenus:2X Linker assembled into fibrillar condensates, while mVenus:FL_Proline remained diffuse (Figure 7a, Figure S6A,B).

This pattern of condensation matches our in vitro observations, where disruption of terminal regions results in a loss of gelation, while tandem duplication of the linker region results in gelation at even lower concentrations than the wildtype protein (Figure 3a,b and 4a,b). Quantification of condensate formation showed robust statistical increases in fiber formation only in CAHS D variants that form gels in vitro (Figure S6A,B). These data indicate that the same sequence features driving in vitro gelation of CAHS D are responsible for its in vivo condensation during osmotic stress.

### 2.9 CAHS D promotes survival during osmotic shock in HEK cells

Since the gelled state of CAHS D has been shown to modulate its protective capacity under different types of desiccation stress (Hesgrove et al., 2021; Packebush et al., 2023), we wondered if gelation of CAHS D in cells during osmotic shock might provide some degree of increased protection. To assess this, we utilized two different cellular survival assays on osmotically stressed cells expressing our mVenus, our wildtype CAHS D protein, and our gelling (2X Linker) and non‐gelling (FL_Proline) variants (Figure S6C,D).

After 24 h of 0.5 M sorbitol osmotic shock, we observed a large decrease in cell viability for mVenus‐expressing cells (Figure 7b, Figure S6C–E). Cells expressing FL_Proline or 2X Linker survived significantly better than mVenus alone (Figure 7b). However, CAHS D retained the highest viability, which was significantly higher than cells expressing mVenus as well as our gelling and non‐gelling variants of CAHS D (2X Linker and FL_Proline) (Figure 7b, Figure S6C–E).

To ensure that the increase in survival seen in osmotically shocked cells expressing CAHS D and its variants is not due a cellular response from an induced ER Stress or unfolded protein response (UPR), we measured the level of ER stress/UPR on the cell lines using a ratiometric stress sensor (Harlen et al., 2019). Expression of mVenus:CAHS D or any of its variants does not induce significant levels of ER stress/UPR compared to naive HEK cells or HEK cells expressing mVenus alone (Figure S6F).

We also checked the corrected total cell green fluorescence from images (Figure S6K) and the green fluorescence intensity (by plate reader, Figure S6L) of the cell lines to estimate the relative amount of protein expression among the variants. All our CAHS D variants show no significant difference in fluorescence, meaning that the cell lines expressing the CAHS D variants are not making wildly different amounts of proteins.

While wildtype CAHS D was significantly more protective than either of its variants, the observation that all CAHS D variants used in this study resulted in increased cellular survival during osmotic shock, regardless of their ability to form gels, suggests that CAHS proteins are protective in both their condensed and uncondensed state in vivo.

### 2.10 Condensed CAHS D serves as a stress‐induced cytoskeleton to preserve cellular ultrastructure, but this does not correlate with increased survival during osmotic stress

The observation that regardless of their condensed state CAHS proteins are protective in cells during osmotic shock intrigued us. This observation is in line with reports that in vitro, CAHS D provides protection in both its soluble and gelled form albeit via distinct mechanisms (Hesgrove et al., 2021; Packebush et al., 2023).

To probe what protective mechanisms might be conferred by condensed CAHS D in cells during osmotic shock, we examined the role of CAHS D condensates in helping maintain cell volume during osmotic shock induced cell shrinkage. This speculation has been prompted by the observation that the mechanism by which CAHS D and other CAHS proteins form gels is in some respects reminiscent of proposed mechanisms underlying Intermediate Filament (IF) formation (Figure 6) (Sysoev et al., 2020; Vermeire et al., 2021; Vermeire & Strelkov, 2023; Zhou et al., 2021), which can stiffen cells (Tanaka et al., 2022). As such, we wondered whether CAHS D might function as a novel type of stress‐inducible cytoskeletal protein, helping to ameliorate the dramatic changes in cell volume associated with hyperosmotic shock.

To assess whether condensation of CAHS proteins can help preserve the structural integrity of cells, we examined volumetric changes associated with osmotic shock in cells expressing gelling and non‐gelling versions of CAHS D. In osmotically shocked cells expressing mVenus alone, we observed a decrease in cell volume of ~50%, which was consistent with cells expressing our non‐gelling mVenus:FL_Proline variant (Figure 7c, Figure S6G). By contrast, in osmotically shocked cells expressing gelling proteins mVenus:CAHS D and mVenus:2X Linker, we observed a reduction in volumetric change of only ~1/3rd, which is significantly less than volume changes observed in mVenus and FL_Proline expressing cells (Figure 7c).

These results suggest that CAHS D, and its variants, are able to better resist osmotically induced volumetric changes, but that this behavior requires the proteins to be in their condensation state.

To assess the degree to which cell viability correlates with the ability of a CAHS protein to reduce volumetric change during osmotic stress, we plotted these values and performed correlation analysis (Figure 7d). While a mild correlation is observed (*R* ^2^ = 0.55), the correlation was not statistically significant. Indicating that while CAHS proteins likely help maintain cell organization and structure during osmotic shock and that this emergent property relies on gelation, it is not correlated with increased survival under osmotic shock.

### 2.11 Gelling variants of CAHS D slow metabolism, which correlates with increased cellular survival, during osmotic shock

The observation that the reduction of volumetric change alone cannot fully explain the protective capacity of CAHS D and its variants during osmotic shock prompted us to examine additional potential mechanisms of protection. In vitro, CAHS D stabilizes enzymes and this is thought to be linked to the ability of CAHS D to slow molecular motions (Figure 1f–h) (Hesgrove et al., 2021; Hesgrove & Boothby, 2020). We wondered if slowed molecular motions and stabilization displayed by CAHS D in vitro carries over to its in vivo function(s) and whether or not CAHS D might be inducing biostasis in cells.

To assess the ability of CAHS D to induce biostasis in cells and the necessity of gelation in this process, we monitored the metabolic activity of cells expressing mVenus, CAHS D, 2X Linker, and FL_Proline during osmotic shock using two different metabolic assays (Figure S6H,I). During osmotic shock, we observed a statistically significant decrease in metabolism in cells expressing our two gelling proteins (CAHS D and 2X Linker) relative to cells expressing mVenus (Figure 7e). By contrast, metabolism of cells expressing non‐gelling FL_Proline was unchanged compared to the mVenus control (Figure 7e). Correlation of metabolic rates with the degree of cellular survival during osmotic shock showed a robust relationship (*R* ^2^ = 0.95) between the degree of reduced metabolism and the survival of stressed cells (Figure 7f).

These results suggest that CAHS D may protect cells through the induction of biostatic slowdown of metabolism, which requires, and is induced by, condensate formation.

### 2.12 Biostasis is reversible in CAHS D expressing cells

In vitro, CAHS D gels are highly soluble and go back into solution rapidly (Figure 1c). A previous report also showed that condensates formed in cells from a different CAHS protein are reversible (Tanaka et al., 2022). We wondered if the same were true for CAHS D and the gelling 2X Linker variant in vivo and, if so, whether the slowing of cell metabolism observed with condensed CAHS D or 2X Linker might also be reversible.

To assess this, we imaged cells recovering from osmotic stress (Figure 7g). Consistent with in vitro results (Figure 1c), mVenus:CAHS D in cells was observed to transition from the condensed state back into solution upon recovery from osmotic shock (Figure 7g). Interestingly, this robust reversal out of the condensation state upon recovery from osmotic shock was much slower for mVenus:2X Linker expressing cells (Figure 7g,h), consistent with a lack of resolubility of the 2X Linker variant in vitro (Figure S5C).

In addition to observing the reversibility of the condensed state of mVenus:CAHS D in cells recovering from osmotic shock, we monitored the reversibility of metabolic depression. While metabolic activity of cells expressing mVenus:CAHS D and mVenus:2X Linker were significantly lower than cells expressing mVenus or mVenus:FL_Proline during osmotic shock (Figure 7e), recovering cells expressing mVenus:CAHS D had metabolisms identical to those of recovering mVenus or mVenus:FL_Proline expressing cells (Figure 7i, Figure S6H–J). However, the metabolism of cells expressing mVenus:2X Linker remained depressed during recovery, mirroring the inability of 2X Linker to decondense (Figure 7i, Figure S6J and S5C). Consistent with the idea that condensation affects metabolism, cells expressing FL_Proline had metabolic rates identical to control cells (Figure 7i, Figure S6J).

Combined, these data show that condensation and associated biostasis (slowing of metabolism) is reversible for wildtype CAHS D, but that this reversibility is compromised in our 2X Linker variant.

## 3 DISCUSSION

CAHS D is an IDP from the tardigrade *H. exemplaris* that is necessary for this animal to successfully survive desiccation‐induced biostasis (Boothby et al., 2017). Here we examine the sequence features and mechanisms underlying the ability of CAHS D to condense into a gel, and the consequences of this condensation for mediating osmotic stress tolerance in cells. We find that the same sequence features that drive in vitro gel formation also promote in vivo condensation of CAHS D. In vivo condensing and non‐condensing forms of CAHS D promote cellular survival, however only the condensed forms help minimize volumetric change and slow metabolism. Interestingly, minimizing volumetric change does not correlate with increased survival during osmotic shock, while lower metabolism does. Finally, just as tardigrades are able to recover from their biostatic state, in cells, CAHS D decondenses upon return to normal osmotic conditions resulting in a return of metabolism to control levels. This study provides insight into how tardigrades, and potentially other desiccation‐tolerant organisms, survive drying by making use of biomolecular condensation. Beyond stress tolerance, our findings provide an avenue for pursuing technologies centered around the induction of biostasis in cells and even whole organisms to slow aging and enhance storage and stability.

### 3.1 Mechanism of CAHS D gel and condensate formation

Our observations that CAHS D forms gels in vitro and in vivo are in line with previous reports (Tanaka et al., 2022; Yagi‐Utsumi et al., 2021). In vitro, gel formation is concentration dependent and in vivo can be prompted through the hyperosmotic shock of cells. We envision that hyperosmotic shock results in a rapid loss of intracellular water, thus concentrating CAHS D and promoting its intracellular self‐association.

Previous reports on the fibrilization and gelation of CAHS proteins have focused mainly on mechanisms exclusively involving the association of helices found within these proteins (Malki et al., 2022; Tanaka et al., 2022; Yagi‐Utsumi et al., 2021), while one group has suggested the involvement of β‐sheets in this process (Eicher et al., 2022; Wang et al., 2023). Here we combine molecular, biochemical, and biophysical experiments with computational analyses of CAHS D to reveal that while its Linker Region is required for gelation it is not sufficient. Rather, all three *N*‐terminal, Linker, and *C*‐terminal Regions, are required for gelation of this CAHS protein. Furthermore, mutagenesis (CLN variant), cross‐linking, and predictive modeling suggests that antiparallel association of the linker results in dimers of CAHS D with heterotypic ends to promote further assembly. Indeed, CAHS D variants with homotypic termini do not form gels. Furthermore, the observation that the diameter of gelled fibers formed by wildtype CAHS D and a 2X Linker variant are statistically identical argues against the idea that CAHS D fibrils form through helical stacking.

Delving deeper into the mechanisms underlying gelation of CAHS D, we find that helicity and amphipathicity of the Linker Region is tuned to promote gelation. Also, β–β interactions between the terminal regions of CAHS D increase and are required for gelation. Based on these observations and coupled with predictions, we arrive at the most parsimonious model of gelation given the current data. In this model, antiparallel association of Linker Regions mediates the association of two CAHS D molecules resulting in a staggered dimer with heterotypic ends. These dimers associate further into bundled structures with a diameter of ~9.5 nm before associating further via end‐to‐end polymerization mediated by β–β interactions (Figure 5e).

Importantly, measurements of fiber diameter from three different experiments (SAXS, AFM (Malki et al., 2022), and SEM) are in good agreement that CAHS D fibers have diameters of ~9–9.5 nm. While SEM imaging of CAHS D and 2X Linker show gel fibers with diameters of ~12 nm, it should be remembered that SEM is known to minimally add ~2 nm to surface thickness (Höflinger, 2013). These measurements of fiber thickness inform our model of how dimers of CAHS D could come together to form such structures. However, equally important as the geometry of the surface of the fibers are the internal dimensions. SAXS analysis on CAHS D gels measures a slight change in the average size of internal void spaces within a fiber from 2.6 to 2 nm as the gel condenses. The presence of mesh structure within the fiber could be of importance, since filling a space with partially hollow fibers is more efficient than doing so with solid assemblies. It should be noted that some morphological differences do appear to manifest in gels produced from CAHS D versus 2X Linker protein. SEM imaging of these gels reveals fibers with a qualitatively smoother morphology relative to wildtype CAHS D (Figure S5A). Similarly, fluorescence imaging reveals qualitatively narrower and longer foci in 2X Linker expressing cells relative to CAHS D expressing cells (Figure 7a,g). We speculate that the longer internal linker of our 2X Linker variant provides a more stable and longer interface for dimer formation relative to wildtype CAHS D. Additionally, the terminal regions, which are likely more bulky than the linker portion of a CAHS fiber, will be more spread out in a gel formed by 2X Linker relative to wildtype CAHS D proteins. This could result in longer and “cleaner” looking fibers.

Interestingly, an earlier study focusing on CAHS 3 from *Ramazzottius varieornatus* used a domain‐bashing approach to identify the minimal sequence required for gelation of this CAHS protein (Tanaka et al., 2022). This analysis revealed that an internal coiled‐coil region flanked by two elements, Region 3 and CR2 are required for fibrillization of CAHS 3 (Tanaka et al., 2022). This fibrillization was speculated to rely exclusively on helical interactions within this minimal sequence (Tanaka et al., 2022), however we found that structural prediction shows a dumbbell‐like ensemble for CAHS 3 strikingly similar to that of CAHS D, with the terminal regions, Region 3 and CR2, containing a significant amount of β‐structure just as in the case of CAHS D (Figure S5E). While we do not discount the involvement of helical interactions in mediating CAHS 3 fibrillization (as seen in CAHS D), the previous study did not address the role of β‐structure within Regions 3 and CR2 (Tanaka et al., 2022). Here we have demonstrated empirically the necessity of β‐structure in mediating CAHS D fibrillization. Thus, based on the structural similarities between CAHS D and the CAHS 3 minimal sequence (Figure S5E), we speculate that a coiled‐coil domain flanked by regions containing β‐structure may be a universal feature required for fibrillization and gelation of CAHS proteins.

### 3.2 Do CAHS gels represent a novel, stress‐inducible cytoskeletal network?

By its most basic definition the cytoskeleton is a system of intracellular filaments or fibers that helps maintain a cell's shape and/or organization (Hohmann & Dehghani, 2019). Typically actin filaments, microtubules, and intermediate filaments are considered the three major classes of cytoskeletal components, but other cytoskeletal components exist, such as the filamenting temperature‐sensitive mutant Z (ftsZ) protein, a prokaryotic functional homolog of tubulin involved in cytokinesis during cell division or septins, which have been dubbed as the “fourth component of the cytoskeleton” (Bisson‐Filho et al., 2017; Mostowy & Cossart, 2012). Other common attributes of cytoskeletal networks are that they are polymers and often made from (homo)monomers (e.g., f‐actin or β‐tubulin) and that they can be dynamically assembled and disassembled (Hohmann & Dehghani, 2019).

Our proposed model of gelation shares similarities to that proposed for intermediate filaments (Figure 6a). In intermediate filament assembly, two copies of an intermediate filament protein form parallel coiled‐coil dimers, and two of these associate to form staggered antiparallel tetramers. Eight such tetramers then assemble into a bundle, which supercoils to form a unit‐length filament (ULF) with a diameter of ~8–10 nm (Figure 6a). These ULFs then further polymerize through end‐to‐end interactions between homotypic headgroups to form intermediate filaments (Alberts, 2017; Hohmann & Dehghani, 2019).

The similarities between CAHS D fibers and intermediate filaments extend beyond the mechanism of their formation, as pointed out previously (Tanaka et al., 2022). Indeed, there appear to be functional similarities between CAHS D fibers and intermediate filaments as well (Figure 6). Intermediate filaments play diverse roles in cells, including the maintenance of cellular stiffness, organization, and integrity (Alberts, 2017; Hohmann & Dehghani, 2019). Here we show that CAHS condensates help maintain the volumetric integrity of cells during osmotic shock (Figure 7c, Figure S6G). In addition, a previous report demonstrates that CAHS proteins help mediate cellular stiffening (Tanaka et al., 2022). However, this report did not test whether gelation is required for stiffening (e.g., would a non‐gelling variant of a CAHS protein still stiffen cells under osmotic stress), nor did it look at the connection between increased cellular survival and stiffening. Here we show that the ability of CAHS D to maintain cell volume is indeed linked to the ability of the protein to gel. However, we demonstrate that there is no significant correlation between the maintenance of cell volume and survival during osmotic stress.

While intermediate filaments are typically thought of as less dynamic than actin filaments and microtubules there are examples of highly dynamic intermediate filament processes (Helfand et al., 2004). As their name implies, intermediate filaments were first distinguished from other cytoskeletal networks in that the fibers they form have an *intermediate* diameter (~10 nm), between that of actin filaments (7 nm) and microtubules (25 nm) (Steinert et al., 1984). Given that CAHS proteins do not share sequence homology with bona fide intermediate filament proteins, but do share similarities in terms of assembly mechanism, dimensions, and function, we take the conservative view that CAHS D condensates represent a novel intermediate filament‐like cytoskeletal network. Importantly, CAHS condensation is dynamic, inducible, and reversible, making it distinct from the current models for intermediate filament assembly. It is of note that other, non‐cytoskeletal, proteins such as the bacterial polar organizing protein Z (PopZ), form assemblages through similar mechanisms, thus these mechanisms of assembly are not exclusive to structural functionality (Bowman et al., 2010; Goodsell & Lasker, 2023).

### 3.3 CAHS D gelation as a mechanism of inducing biostasis

Induction of biostasis, the slowing down or halting of biological processes, has long been implicated in desiccation tolerance. This idea is encapsulated by both the molecular shielding (Boothby & Pielak, 2017; Goyal, 2005; Hesgrove & Boothby, 2020) and vitrification (Boothby & Pielak, 2017; Crowe et al., 1998; Hesgrove & Boothby, 2020) hypotheses. In the first, protectants act as molecular shields, getting in the way of aggregation prone proteins slowing or altogether preventing their interactions (Boothby, 2019; Goyal, 2005; Hesgrove et al., 2021; Hesgrove & Boothby, 2020). In the second, a super‐viscous state is thought to be induced that ultimately forms an amorphous (vitrified) solid, preventing both large and small scale molecular motion such as diffusion of molecules and protein unfolding (Boothby & Pielak, 2017; Crowe et al., 1998; Hengherr et al., 2009; Hesgrove & Boothby, 2020; Sakurai et al., 2008). While vitrification is not sufficient to confer protection during desiccation (many non‐protective molecules are known to vitrify) it is generally accepted as a requirement to survive in the dry state (Crowe et al., 1998). CAHS proteins, as well as tardigrades, have been shown to vitrify (Boothby, 2021; Hengherr et al., 2009), which is not surprising given the capacity of CAHS proteins to be disordered and to form gels, which themselves are often amorphous solids (Douglas, 2018; Horkay et al., 2019).

Importantly, gels are viscoelastic materials. We envision that the viscous component of CAHS gels helps slow the diffusion and motion of metabolites, similarly, super viscous states induced by gelation may help reduce diffusion, aggregation, and/or relaxations of proteins. The elastic component of CAHS gels may also serve a role(s) in stress tolerance, for example the preservation of cellular ultrastructure. While we did not observe a strong correlation between reduced cell shrinkage and survival during hyperosmotic shock, it is possible that elastic properties of CAHS gels are important under more severe water stresses.

Our observation that reduced cellular metabolism is a feature of osmotically shocked cells expressing variants of CAHS D that can condense (in vivo) or gel (in vitro) and that in vivo this correlates strongly with survival, suggests that induction of biostasis maybe a mechanism underlying CAHS D mediated protection and is specific to the protein's capacity to form condensates. Notably, while osmotically stressed cells expressing our 2X Linker variant do show slowed metabolism, returning these cells to normal osmotic conditions does not lead to recovered metabolism. This continued depression of metabolic rates in these cells is mirrored by the 2X Linker protein's diminished capacity to resolvate both in vitro and in vivo (Figure S5C, Figure 7g,h).

It is of note that a non‐condensing variant (FL_Proline) of CAHS D also provides statistically significant protection to human cells undergoing hyperosmotic stress. This variant does not slow metabolism, which suggests that CAHS D is capable of conferring protection against hyperosmotic stress at the cellular level in both in condensed and uncondensed form, likely through distinct mechanisms. This is in line with previous in vitro studies which show that gelling and non‐gelling variants of CAHS D protect labile proteins to varying degrees during desiccation (Biswas et al., 2024; Hesgrove et al., 2021; Packebush et al., 2023). In its non‐condensed form, CAHS D might confer protection to cells undergoing hyperosmotic stress through the prevention of protein aggregation via molecular shielding and/or stabilization of membranes via helical absorption (Hesgrove & Boothby, 2020).

Overall, our current study provides the first empirical evidence for a condensation‐specific role for CAHS D: the induction of reversible biostasis during osmotic shock. This work advances our fundamental knowledge of how tardigrades survive extreme abiotic stress by providing a holistic and mechanistic model for the formation of a novel stress‐inducible cytoskeleton composed of CAHS D, which promotes cellular survival not through the maintenance of cell volume, but rather through the induction of reversible biostasis. Beyond tardigrades, our work provides avenues for pursuing novel cell‐stabilization/anti‐aging technologies as well as provides insights into how CAHS proteins from tardigrades might be used and/or engineered to stabilize sensitive pharmaceuticals.

## 4 METHODS

### 4.1 Cloning

All variants and wild type CAHS D except GS variants were cloned into the pET28b expression vector using Gibson assembly methods. Primers were designed using the NEBuilder tool (NEB), inserts were synthesized as gBlocks and purchased from Integrated DNA Technologies (IDT). Clones were propagated in DH5α cells available from NEB (Catalog C2987H).

GS variants were designed by replacing the Linker region of CAHS D with glycine‐serine (GS) repeats of 25, 53, and 105 amino acids. The constructs were cloned after NotI in pMalC6T (NEB) by Twist Bioscience. Clones were propagated in DH5α cells available from NEB (Catalog C2987H).

No hydro, NAH, and RAH sequences were designed using sequence analysis tools built into sparrow (https://github.com/idptools/sparrow/) and GOOSE (https://github.com/idptools/goose).

### 4.2 Protein expression

Expression constructs were transformed in BL21 (DE3) *E*. *coli* (Catalog C2527H, NEB) and plated on LB agar plates containing 50 μg/mL Kanamycin for constructs in pET28b and containing 100 μg/mL Ampicillin for constructs in pMalC6T. At least three single colonies were chosen for each construct and tested for expression.

Large‐scale expression for constructs in pET28b was performed in 1 L LB/Kanamycin cultures, shaken at 37°C (Innova S44i, Eppendorf) until an OD 600 of 0.6, at which point expression was induced using 1 mM IPTG. Protein expression was continued for 4 h, after which cells were collected at 4000*g* for 30 min at 4°C. Cell pellets were resuspended in 5 mL of resuspension buffer 20 mM tris, pH 7.5, 30 μL protease inhibitor (Catalog P2714‐1BTL, Sigma Aldrich). Pellets were stored at −80°C.

Large‐scale expression for constructs in pMalC6T was performed in 1 L LB/Ampicillin supplemented with 0.2% glucose cultures, shaken at 37°C (Innova S44i, Eppendorf,) until an OD600 of 0.5, at which point expression was induced using 0.3 mM IPTG. Protein expression continued for 4 h, after which cells were collected at 4000*g* for 30 min at 4°C. Cell pellets were resuspended in 10% w/v of resuspension buffer 20 mM tris, 200 mM NaCl, 1 mM EDTA, pH 7.4, 30 μL protease inhibitor (Catalog P2714‐1BTL, Sigma Aldrich). Resuspended pellets were stored at −80°C until further use.

### 4.3 Protein purification

Purification for constructs in pET28b largely follows the methods in Piszkiewicz et al. (2019). Bacterial pellets were thawed and heat lysis was performed. Pellets were boiled for 10 min and allowed to cool for 10 min. All insoluble components were removed via centrifugation at 13,000*g* at 10°C for 30 min. The supernatant was sterile filtered with 0.45 and 0.22 μm syringe filters (Catalog 388‐3416‐OEM, EZFlow Syringe Filter). The filtered lysate was diluted 1:2 in purification buffer UA (8 M Urea, 50 mM sodium acetate, pH 4). The protein was then purified using a cation exchange HiPrep SP/HP 16/10 (Cytiva) on the AKTA Pure 25 L (Cytiva), controlled using the UNICORN 7 Workstation pure‐BP‐exp (Cytiva). Variants were eluted using a gradient of 0%–50% UB (8 M Urea, 50 mM sodium acetate, and 1 M NaCl, pH 4), over 20 column volumes.

Fractions were assessed by SDS‐PAGE and pooled for dialysis in 3.5 kDa MWCO dialysis tubing (SpectraPor 3 Dialysis Membrane, Catalog 132724, Sigma Aldrich). For all variants in pET28b except CLC, pooled fractions were dialyzed at 25°C for 4 h against 2 M urea, 20 mM sodium phosphate at pH 7.0, then transferred to 20 mM sodium phosphate at pH 7 overnight. This was followed by six rounds of 4 h each in Milli‐Q water (18.2 MΩcm). Dialyzed samples were quantified fluorometrically (Qubit 4 Fluorometer, Catalog Q33226, Invitrogen), aliquoted in the quantity needed for each assay, and lyophilized (Catalog 7752021, Labconco FreeZone 6) for 48 h, then stored at −20°C until use. CLC was dialyzed in 2 M urea, 20 mM Tris at pH 7 for 4 h, followed by 6 rounds of 4 h each in 20 mM Tris pH 7. CLC samples were quantified using Qubit 4 fluorometer as described, concentrated using spin‐concentrators (Catalog UFC900324, Millipore‐Sigma) to the desired concentration and used immediately.

Purification of the GS constructs in pMalC6T were performed following the NEB NEBEXpress MBP Fusion and Purification System protocols (Catalog E8201S, NEB). Pellets were thawed in cold water and lysed by sonication for 10 min, using 40% amplitude and 30 s on/30 s off cycles on ice. All insoluble components were removed via centrifugation at 13,000*g* at 10°C for 30 min. The supernatant was filtered with 0.45 and 0.22 μm syringe filters (EZFlow Syringe Filter, Cat. 388‐3416‐OEM). The proteins were then purified by affinity chromatography using amylose resin (Catalog E8021L, NEB). The amylose resin was washed and pre‐equilibrated in 20 mM tris, 200 mM NaCl, 1 mM EDTA, pH 7.4 buffer before the supernatant from the cell extract was loaded. After the supernatant was loaded the resin was washed with 12 column volumes of 20 mM tris, 200 mM NaCl, 1 mM EDTA, pH 7.4 buffer.

MBP and the GS linkers are fused by a polylinker containing a TEV protease recognition site for easy removal of the MBP‐tag. Cleavage of GS linkers from MBP was done in column using 1 unit of TEV protease (Catalog P8112S, NEB) for every 2 μg of fusion protein in 1X TEV buffer (Catalog P8112S, NEB) for 16 h at 30°C.

GS proteins were eluted as flow‐through, using two column volumes of 20 mM tris, 200 mM NaCl, 1 mM EDTA, pH 7.4 buffer to wash off the proteins from the resin.

TEV Protease and MBP contain polyhistidine tags at their *N*‐termini and they can be removed from the cleavage reaction by immobilized metal affinity chromatography, such as Nickel or Cobalt resin, TEV protease and any free MBP or uncleaved MBP fusion were further removed using a cobalt resin (Catalog 786‐402, G‐Biosciences).

Purified GS proteins were assessed by SDS‐PAGE and pooled for dialysis in 3.5 kDa MWCO dialysis tubing (SpectraPor 3 Dialysis Membrane, Catalog 132724, Sigma Aldrich). Proteins were dialyzed at 25°C for 4 h against 25 mM Tris–HCl, 150 mM NaCl, pH 7.4, then transferred to 25 mM Tris–HCl, 50 mM NaCl, pH 7.4 overnight. This was followed by four rounds of 4 h each in Milli‐Q water (18.2 MΩcm). Dialyzed samples were quantified fluorometrically (Qubit 4 Fluorometer, Catalog Q33226, Invitrogen), aliquoted in the quantity needed for each assay and lyophilized (Catalog 7752021, Labconco FreeZone 6) for 48 h, then stored at −20°C until use.

### 4.4 Visual assay for concentration and temperature dependence of gelation

Quantitated and lyophilized protein samples were transferred as powder into NMR tubes (Catalog WG‐4001‐7, Bel‐Art Products) and resuspended in 500 μL of water to final concentrations of 5, 10, 15, and 20 g/L. Samples were left at room temperature for 5 min to solubilize. If solubilization was not occurring (as determined visually), samples were moved to 55°C for intervals of 5 min until solubilized. If solubilization was not progressing at 55°C after 10 min of heating (as determined visually), then samples were transferred 95°C for 5‐min intervals until fully solubilized.

Solubilized proteins were transferred from heat blocks to the bench and left at ambient temperature for 1 h. Tubes were then loaded horizontally into a clamp holder and photographed. Gelation was visually assessed by the degree of solidification or flow of the sample in the NMR tube.

### 4.5 Visual assay for gel resolubility assessment

After 1 h at ambient temperature, proteins that had been found to form gels were transferred to a 55°C heat block for 1 h to thermally resolubilize them. After an hour, samples were returned to the photographic clamp holder and imaged immediately to confirm that gelation had been disrupted. Samples were placed upright on the bench at ambient temperature for 1 h to gels reform upon cooling to room temperature.

To assess resolubility, samples of 10 g/L CAHS D and 2X LR were prepared as described above. Buffer (Tris, 20 mM pH 7) was added to the gels to bring the final concentration of solvated CAHS D and 2X LR to 2.5 g/L, which is below the gelation point of both proteins. Samples were photographed before and immediately after addition of buffer, vortexed for 5 s, and left to resolubilize. For heat resolubilization, resolvated samples were moved to a 55°C heat block and the time for solubilization was recorded.

### 4.6 Scanning electron microscopy, critical point drying, and image analysis

Protein samples were heated to 95°C for 5 min and 50 μL of each sample were transferred to microscope coverslips. Samples were fixed in a 2.5% glutaraldehyde/4% paraformaldehyde solution for 10 min. Samples were then dehydrated in an ethanol series going from 25%, 50%, 75%, 3× 100% with each incubation time being 10 min. Dehydrated samples were prepared for imaging using critical point drying (Tousimis Semidri PVT‐3, SGC Equipment) and supporter coating (108 Auto, Cressington Scientific Instrument). Imaging was performed on a Hitachi S‐47000 scanning electron microscope.

Image analysis for fiber diameter and ULF lengths was carried out using FIJI (V. 2.3.0/1.52q).

### 4.7 Lactate dehydrogenase protection assay

LDH desiccation protection assay was performed in triplicate as described previously (Boothby et al., 2017; Piszkiewicz et al., 2019). Briefly, CAHS D was resuspended in a concentration range from 20 g/L to 0.1 g/L in 100 μL resuspension buffer (25 mM Tris, pH 7.0). Rabbit Muscle L‐LDH (Catalog 10127230001, Sigma‐Aldrich) was added to this at 0.1 g/L. Half of each sample was stored at 4°C, and the other half was desiccated for 17 h without heating in a speedvac (OFP400, Thermo Fisher Scientific). Following desiccation all samples were brought to a volume of 250 μL with water. The LDH/CAHS D mixture was added 1:10 to the assay buffer (100 mM Sodium Phosphate, 2 mM Sodium Pyruvate, 1 mM NADH, pH 6). Enzyme kinetics were measured by NAD^+^ absorbance at 340 nm, on the NanodropOne (Thermo Fisher Scientific). The protective capacity was calculated as a ratio of NAD^+^ absorbance in desiccated samples normalized to non‐desiccated controls. Sigmoidal curve has been added as a visual guide.

### 4.8 FTIR sample preparation and measurement

All samples were prepared by dissolving the CAHS D protein in D_2_O to avoid the overlapping of the amide I band of CAHS D (centered at 1650 cm^−1^) with the H_2_O bending mode (around 1640 cm^−1^) (Maréchal, 2011). Gel samples were obtained by dissolving the lyophilized CAHS D protein at a concentration of 16 g/L in D_2_O heated at about 50°C and gently stirred for 5 min. A volume of 10 μL of the CAHS D solution was deposited between two CaF_2_ windows, mounted into a Jasco MagCELL sample holder. Glassy samples were obtained by depositing a 38 μL drop of the heated CAHS D solution (16 g/L) in D_2_O on a 50 mm diameter CaF_2_ window. The sample was dried under N_2_ flow for 5 min and subsequently the optical window on which the glass had formed was inserted into a sample holder equipped with a second CaF_2_ window to form a gas‐tight cylindrical cavity (Malferrari et al., 2011). Different hydration levels of the CAHS D glass were obtained by an isopiestic method, that is, by equilibrating the sample with saturated salt solutions providing defined values of relative humidity (RH), contained at the bottom of the sample holder cavity. The following saturated solutions in D_2_O were employed to obtain the desired RH (Greenspan, 1977) at 297 K: KNO_3_ (RH = 95%), NaCl (RH = 75%), Mg(NO_3_)_2_ (RH = 53%), K_2_CO_3_(RH = 43%), MgCl_2_ (RH = 33%), CH_3_COOK (RH = 23%), and LiCl (RH = 11%).

FTIR absorption measurements were performed at room temperature with a Jasco Fourier transform 6100 spectrometer equipped with a DLATGS detector. The spectra, in the range (7000–1000 cm^−1^) with a resolution of 2 cm^−1^, were acquired by averaging 10^3^ interferograms, using a standard high‐intensity ceramic source and a Ge/KBr beam splitter.

### 4.9 Amide hydrogen/deuterium exchange kinetics in a CAHS D dried matrix at different hydration levels

Amide H/D exchange can be followed by FTIR spectroscopy of the amide II band of proteins centered around 1550 cm^−1^ in H_2_O. Following H/D exchange the wavenumber of this mode is downshifted at 1450 cm^−1^ (Barth, 2007) (the so‐called amide II' band) Thus, the two bands are well separated in the spectrum.

Two samples of CAHS D protein in H_2_O (16 g/L) were extensively dried by equilibration within gas‐tight holders at relative humidity RH = 6% in the presence of NaOH·H_2_O, by using the isopiestic method described in the FTIR Sample Preparation and Measurement section. After equilibration, H/D exchange at RH = 11% was started in one of the samples by placing saturated LiCl in D_2_O in the sample holder to create an 11% D_2_O atmosphere. H/D exchange at RH = 75% was begun in the second sample by placing a saturated solution of NaCl in D_2_O in the holder. It has been previously shown (Malferrari et al., 2012; Malferrari, Francia, et al., 2016) that such an isopiestic approach for isotopic exchange is quite effective and rapid (hour time scale). In order to obtain the kinetics of H/D exchange (Figure 1g, Figure S1E), a series of FTIR spectra was then recorded in sequence, at selected time intervals following the start of H/D exchange. For each spectrum, only 100 interferograms were averaged to allow a sufficiently rapid acquisition (3 min). We evaluated the extent of amide H/D exchange from the area of the amide II' band at 1450 cm^−1^ after subtraction of a straight baseline drawn between the minima on either side of the band (Goormaghtigh et al., 1994).

### 4.10 Measurement of conformational dynamics of photosynthetic reaction centers embedded in CAHS D glasses

The photosynthetic reaction center (RC) from the purple bacterium *Rhodobacter* (*Rb*.) *sphaeroides* represents an ideal model system to probe matrix effects on conformational protein dynamics. This membrane‐spanning pigment‐protein complex catalyzes the primary photochemistry of bacterial photosynthesis. Following photoexcitation, the primary electron donor (P) of the RC (a bacteriochlorophyll dimer situated near the periplasmic side of the protein) delivers an electron in about 200 ps to the primary quinone acceptor, Q_A_ (located 25 Å away from P and closer to the RC cytoplasmic side) thus generating the primary charge separated state, P^+^Q_A_^−^ (Figure S1F). In the presence of o‐phenanthroline, (an inhibitor which blocks electron transfer from Q_A_^−^ to Q_B_), the electron on Q_A_^−^ recombines with the hole on P^+^ by direct electron tunneling (Feher et al., 1989).

The kinetics of P^+^Q_A_^−^ recombination after a short (ns) flash of light provides an endogenous probe of the RC conformational dynamics. In fact, when the RC relaxation from the dark‐adapted to the light‐adapted conformation is hampered (e.g., at cryogenic temperatures (McMahon et al., 1998), or by incorporation of the RC into dehydrated trehalose glasses (Palazzo et al., 2002)) the recombination kinetics are accelerated and become strongly distributed, mirroring the immobilization of the protein over a large ensemble of conformational substrates. Accordingly, charge recombination kinetics of confined RCs are described by a continuous distribution *p*(*k*) of rate constants *k* (Palazzo et al., 2002) (Figure 1h, Figure S1G,H):

(1) $$N\left(t\right)=\int_{0}^{\infty }p\left(k\right)\exp\left(-\mathrm{kt}\right)\mathrm{dk}={\left(1+\frac{\sigma^{2}}{<k>}t\right)}^{-\frac{<k{>}^{2}}{\sigma^{2}}}$$

where *N*(*t*) is the survival probability of the P^+^Q_A_^−^ state at time t after photoexcitation, <*k>* is the average rate constant, and *σ* is the width of the rate distribution *p*. An increase in either or both parameters (<*k>*, *σ*) reflects inhibition of the RC internal dynamics, that is, slowing of relaxation from the dark‐ to the light‐adapted conformation (<*k>*), or of the interconversion between conformational substates (*σ*) (Malferrari et al., 2015; Palazzo et al., 2002).

CAHS D: RC glassy samples were prepared by mixing a 78 μL volume of 76 μM RC purified from *Rb. sphaeroides* R26 in assay buffer (10 mM Tris, 0.025% LDAO, pH 8.0) with 64 μL of 16 g/L CAHS D protein in water, and 8 μL of 200 mM o‐phenanthroline in ethanol. This mixture was deposited at the center of a 50 mm diameter CaF_2_ optical window and solid samples were obtained and equilibrated at RH by using the isopiestic approach described in the *FTIR Sample Preparation and Measurement* section.

The kinetics of P^+^Q_A_^−^ recombination after a laser pulse (Figure 1h) were measured by time‐resolved optical absorption spectroscopy, by recording the absorbance change at 422 nm, essentially as described in (Francia et al., 2008). At each RH tested the residual water content of the CAHS D: RC matrices was determined from the area of the (υ_2_+υ_3_) combination band of water at 5155 cm^−1^ (Malferrari et al., 2011; Malferrari et al., 2015). The water content of the matrices has been expressed as the mass ratio of water: dry matrix. To determine the mass of the dry matrix, we included the CAHS D protein, the RC, and the detergent belt of the RC formed by 289 LDAO molecules (Malferrari et al., 2011) plus 14 molecules of free LDAO per RC. The hydration isotherms obtained for dried CAHS D: RC and trehalose: RC matrices are essentially coincident and well fit the Hailwood and Horrobin equation (Hailwood & Horrobin, 1946) (Figure S1I).

### 4.11 Proteome‐wide bioinformatics

The tardigrade proteome (tg.default.maker.proteins.fasta), taken from https://github.com/sujaikumar/tardigrade was used. The proteome file was pre‐processed using protfasta (https://protfasta.readthedocs.io/), IDRs predicted with metapredict (Emenecker et al., 2021) and IDR kappa values calculated using localCIDER (Das & Pappu, 2013; Holehouse et al., 2017). Metapredict identified 35,511 discrete IDRs distributed across 39,532 proteins in the tardigrade proteome.

### 4.12 All‐atom simulations

All‐atom simulations were run using the ABSINTH implicit solvent model and CAMPARI Monte Carlo simulation engine (https://campari.sourceforge.net/). ABSINTH simulations were performed with the ion parameters derived by Mao and Pappu (2012). The combination of ABSINTH and CAMPARI has been used to generate experimentally‐validated ensembles for a wide range of IDRs (Martin et al., 2016, 2020; Metskas & Rhoades, 2015).

Simulations were performed for the full‐length CAHS D protein starting from a randomly generated non‐overlapping starting state. Monte Carlo simulations update the conformational state of the protein using moves that perturb backbone dihedrals, and sidechain dihedrals, and rigid‐body coordinates of all components (including explicit ions).

Ten independent simulations were run for 150,000,000 steps each in a simulation droplet with a radius of 284 Å at 310 K. The combined ensemble generated consists of 69,500 conformations, with ensemble average properties computed across the entire ensemble where reported. Finally, we subsampled to analyze one in every 10 frames, providing us with an ensemble of 6950 frames total. All simulation analyses presented here were performed on this smaller ensemble of 6950 conformers, although equivalent results are obtained if the full 69,500 conformer ensemble is used. We reduced the ensemble size down to 6950 to make Bayesian reweighting via SAXS curves tractable, as to re‐weight, each conformer in the ensemble must have a scattering curve calculated (discussed below).

Given the size of the protein, reliability with respect to residue‐specific structural propensities is likely to be limited, such that general trends should be taken as a qualitative assessment as opposed to a quantitative description. We share this caveat especially in the context of future re‐analysis of our simulations. Simulations were analyzed using SOURSOP and MDTraj (Lalmansingh et al., 2023; McGibbon et al., 2015). Simulated scattering profiles were generated using FOXS (Schneidman‐Duhovny et al., 2013).

### 4.13 Polymer models

In addition to performing all‐atom simulations, in Figure 2 we reported distributions for the radius of gyration for two reference states: the Analytical Flory Random Coil (AFRC) and the self‐avoiding random coil (SARC), also previously referred to as the Excluded Volume (EV) simulation in our previous work.

The AFRC model generates a sequence‐specific Gaussian chain parameterized on all‐atom simulations to generate an ensemble that matches the expected dimensions if CAHS D behaved like a Gaussian chain (Alston et al., 2023). The SARC model is a numerically‐generated ensemble obtained by performing all‐atom simulations in which all interactions other than the repulsive component of the Lennard‐Jones potential are turned off, resulting in a sequence‐specific model of the excluded volume accessible to a polypeptide. For more details on the SARC model, see Holehouse et al. (2015), where we refer to it as the EV ensemble.

### 4.14 Bayesian reweighting of simulations

The subsampled simulation ensemble (6950 frames) was reweighted by the Bayesian/Maximum‐Entropy (BME) method using a Python script from Bottaro and colleagues (https://github.com/KULL-Centre/BME) (Bottaro et al., 2020). Per‐frame scattering curves were calculated from simulation data using FOXS (Schneidman‐Duhovny et al., 2013). The goodness of fit between the calculated and experimental SAXS curves prior to reweighting is described by an initial reduced chi‐squared (*χ* ^2^) value of 11.25. The parameter *θ* is a scaling factor such that a lower *θ* value should yield a lower reweighted *χ* ^2^ (better agreement with experimental data). However, as *θ* and *χ* ^2^ decrease, so does the fraction of effective simulation frames (*Φ*) used in the reweighted ensemble. In this case, *χ* ^2^ was largely unchanged at *θ* values of <100, so we set *θ* = 50 to maximize the fraction of simulation frames reflected in the reweighted ensemble.

The reweighted ensemble shows a much better agreement with the SAXS data (*χ* ^2^ = 1.71) and preserves ~46% of the per‐frame simulation data (*Φ* = 0.46). Note that no simulation frames are discarded in the reweighted ensemble, but the BME protocol gives each frame a new weight such that the sum of the weights of all frames equals 1. For ensuing simulation analysis using the reweighted ensemble, the per‐frame weights were applied when calculating ensemble‐averaged features including the radius of gyration, intramolecular distances, and secondary structure.

### 4.15 Contact order analysis

The contact order quantifies the fraction of the simulation in which each residue is in direct contact with any other backbone or sidechain. Contact here is defined as two heavy atoms under 5 Å. We calculated the contact order using SOURSOP with the get_contact_map() function (Lalmansingh et al., 2023).

### 4.16 Small‐angle x‐ray scattering

All SAXS measurements were performed at the BioCAT (beamline 18ID at the Advanced Photon Source, Chicago, IL). SAXS measurements on monomeric CAHS D were collected with in‐line size exclusion chromatography (SEC‐SAXS) coupled to the x‐ray sample chamber to ensure the protein was monomeric. Concentrated protein samples were injected into a Superdex 200 increase column (Cytiva) pre‐equilibrated in a buffer containing 20 mM Tris pH 7, 2 mM DTT, and 50 mM NaCl. Scattering intensity was recorded using a Pilatus3 1 M (Dectris) detector placed 3.5 m from the sample, providing a q‐range from 0.004 to 0.4 Å^−1^. One‐second exposures were acquired every 2 s during the elution. Data were reduced at the beamline using the BioXTAS RAW 1.4.0 software (Hopkins et al., 2017). The contribution of the buffer to the x‐ray scattering curve was determined by averaging frames from the SEC eluent, which contained baseline levels of integrated x‐ray scattering, UV absorbance, and conductance. Baseline frames were collected immediately pre‐ and post‐elution and averaged. Buffer subtraction, subsequent Guinier fits, and Kratky transformations were done using custom MATLAB (Mathworks) scripts or with custom Python code using the BioXTAS RAW 2.1.4 API (https://bioxtas-raw.readthedocs.io/en/latest/).

CAHS D samples were prepared for SAXS measurements by dissolving 5 mg/mL lyophilized CAHS D protein into a buffer containing 20 mM Tris pH 7 and 50 mM NaCl. Samples were incubated at 60°C for 20 min to ensure the sample was completely dissolved. Samples were syringe filtered to remove any remaining undissolved protein before injecting 1 mL onto the Superdex 200 column (Cytiva).

SAXS data for CAHS D gels were obtained by manually centering capillaries containing premade gels in the x‐ray beam. Data were recorded as a series of thirty 0.2 s exposures, but only the first exposure was analyzed to minimize artifacts from x‐ray damage. The final analyzed data were corrected for scattering from the empty capillary and a buffer containing capillary. CAHS D gel‐containing samples were made by dissolving 100 mg/mL lyophilized protein in a buffer containing 20 mM Tris pH 7 and 50 mM NaCl. The sample was incubated for 20 min at 60°C to ensure the protein was completely dissolved. Double open‐ended quartz capillaries with an internal diameter of 1.5 mm (Charles Supper) were used to make the samples. Dissolved protein was directly drawn into the capillary via a syringe. Concentration gradients were generated by layering the protein with buffer. Both ends of the capillary were then sealed with epoxy. Samples were allowed to cool for 5 h prior to measurement. Because of the buffer layering method used to create the concentration gradient in gelled CAHS D samples, the precise concentration of the gel at any given point along the length of the capillary tube is not explicitly known. A concentration gradient can be defined from the bottom to the top of the capillary tube up as high to low CAHS D concentration. Data were collected along the concentration gradient by collecting data in 2 mm increments vertically along the capillary.

All data analysis was done using custom MATLAB (Mathworks) scripts. First, an effective concentration was calculated by assuming the maximum concentration was 100 mg/mL and scaling the remaining samples by the integrated intensity of the form factor. It should be noted that the actual concentration could be significantly less than 100 mg/mL in the maximum concentration sample. Data were fit to an equation containing three elements to describe the features apparent in the scattering data. The high‐angle form factor was modeled using a Lorentzian‐like function to extract the correlation length and an effective fractal dimension.

(2) $$I\left(q\right)=\frac{A_{1}}{1+{\left(\mathrm{q\zeta}\right)}^{d}}$$

The correlation length is given by and is related to the mesh size inside the fiber bundles seen in SEM images. The fractal dimension, 𝑑, is related to the density of the mesh. No clear correlation length was observed in the smallest angle data, and thus a power law was used to account for this component. The exponent, is related to the nature of the interface inside and outside of the bundles.

(3) $$I\left(q\right)\sim A_{2}*q^{-d}$$

Finally, a Lorentzian peak was used to fit the diffraction peak that is apparent at higher concentrations. The width of the peak, B, appeared constant and was thus fixed so that the amplitude could be accurately extracted.

(4) $$I\left(q\right)\sim \frac{A_{3}}{1+{\left|\frac{q-q_{0}}{B}\right|}^{2}}$$

In all fit components, *Ax* is a scale factor.

### 4.17 Ensemble optimization method

Ensemble Optimization Method (EOM) data were generated using ATSAS online's EOM web portal (https://www.embl‐hamburg.de/biosaxs/atsas‐online/eom.php) (Bernadó et al., 2007; Tria et al., 2015). Inputs for the simulation included a buffer subtracted SEC SAXS curve of CAHS D and the amino acid sequence of CAHS D. The terminal regions (residues 1–90 and 196–227) of CAHS D were described as broadly disordered and the linker region (residues 91–195) was described as broadly helical. No distance constraints were used. The average radius of gyration was determined from the EOM ensemble histogram using the following equation:

(5) $$<R_{g}>=\sum R_{g}*P\left(R_{g}\right)$$

In which *R*_g_ is the value and *P*(*R*_g_) is the EOM‐derived probability for that *R*_g_. The reported error is the standard deviation from the above average, calculated according to:

(6) $$\sigma =\sqrt{\sum {R_{g}}^{2}*P\left(R_{g}\right)-{\left[\sum R_{g}*P\left(R_{g}\right)\right]}^{2}\;}$$

### 4.18 Sequence analysis

In Figure 2, sequence analysis was performed using sparrow (https://github.com/idptools/sparrow) and disorder prediction was performed using metapredict (Emenecker et al., 2022). Sequence to radius of gyration predictions from ALBATROSS were performed using the metapredict webserver (https://metapredict.net/), which uses sparrow (https://github.com/idptools/sparrow) (Lotthammer et al., 2023).

### 4.19 CD spectroscopy at non‐gelling concentrations

Lyophilized protein constructs were weighed and dissolved in a 20 mM Tris–HCl (Fisher Bioreagents) buffer at pH 7.0. CD spectra were measured using a JASCO J‐1500 CD spectrometer with 0.1 cm quartz cell (Starna Cells, Inc,) using a 0.1 nm step size, a bandwidth of 1 nm, and a scan speed of 200 nm/min. Each spectrum was measured 7 times and averaged to increase the signal‐to‐noise ratio. The buffer control spectrum was subtracted from each protein spectrum (Figures S2L,M and S3A–D). The resulting spectra were deconvoluted to predict secondary structure contents using the lsq_linear function from the SciPy library. To do this, base spectra for α‐helix, β‐sheet, and random coil spectra (taken from (Perczel et al., 1992)) were linearly fit to match the experimental data set.

### 4.20 CD spectroscopy at gelling concentrations

To prepare stock solutions, freeze‐dried proteins were dissolved in 20 mM MES buffer pH 6.0 and then pH was adjusted to 6.00 ± 0.02 at 25.0 ± 0.1°C using pH Sensor InLab® Ultra‐Micro‐ISM (Mettler Toledo) calibrated before measurement. Gelled proteins were melted at 50°C prior to pH measurement and adjustment, and then equilibrated at 25.0 ± 0.1°C.

CD data were collected on a JASCO J‐810 spectropolarimeter fitted with a Peltier temperature controller (Jasco UK). A quartz cuvette with a light path of 0.1 cm was used. The samples were equilibrated in the CD instrument thermostated at 5°C for 10 min prior to recording full spectra between 190 or 200 and 260 nm with a 1 nm step size, 100 nm min^−1^ scanning speed, 1 nm bandwidth and 1 s response time. Then variable temperature experiments were performed by heating and cooling samples 5–90–5°C at a rate of 30°C/h whilst monitoring CD at 222 nm at 0.5°C intervals followed by full spectra at 5°C.

Baselines recorded using the same buffer, cuvette and parameters were subtracted from each dataset. The spectra were converted from ellipticities (deg) to mean residue ellipticities (MRE, (deg·cm^2^·dmol^−1^·res^−1^)) by normalizing for concentration of peptide bonds and the cell path length using the Equation (7):

(7) $$\mathrm{MRE}\;\left(\deg\cdot {\mathrm{cm}}^{2}\cdot {\text{dmol}}^{-1}\cdot {\mathrm{res}}^{-1}\right)=\frac{\theta *100}{c*l*b}$$

where the variable *θ* is the measured difference in absorbed circularly polarized light in millidegrees, *c* is the millimolar concentration of the specimen, l is the path‐length of the cuvette in cm and *b* is the number of amide bonds in the polypeptide, for which the *N*‐terminal acetyl bond was included but not the *C*‐terminal amide.

Fraction helix (%) was calculated from MRE at 222 nm using the following equation (8):

(8) $$\text{Fraction helix}\;\left(\%\right)=100*\frac{{\mathrm{MRE}}_{222}-{\mathrm{MRE}}_{\text{coil}}}{-42,500*\left(1-\frac{3}{n}\right)-{\mathrm{MRE}}_{\text{coil}}}$$

where MRE_coil_ = 640–645 T; *T* is the temperature in °C; and *n* is the number of amide bonds in the sample (including the *C*‐terminal amide) (Myers et al., 1997).

### 4.21 Helical wheel plot

Helical wheel plots were generated using the heliQuest sequence analysis module. CAHS D linker sequence 126–144 was used, with the α‐helix option chosen as the helix type.

### 4.22 Differential scanning calorimetry (DSC) method

Prior to DSC measurement, samples were prepared in Eppendorf tubes at the desired concentration by adding protein into water. Protein solutions were then vortexed and heated to 55°C for 5 min to ensure proper mixing and that samples were completely solubilized. Forty microliters of solubilized sample was hermetically sealed into a previously massed pair of DSC aluminum hermetic pan and hermetic lid (Catalog 900793.901 and 901684.901, respectively, TA instruments). The sample mass was determined after the sample was sealed within the pan and lid. The sealed samples were then run on a TA DSC2500 instrument. Trios software (TRIOS version #5.0.0.44608, TA Instruments) provided by TA Instruments was used to perform analysis of the DSC data. Details for DSC methods for specific figures are listed below:

Figure 1a DSC Method

The heating run for different concentrations of CAHS D samples consisted of samples being equilibrated at 20°C and then heated to 80°C at a 5°C per minute ramp.

The heating and cooling run consisted of the samples starting at the stand‐by temperature of 20°C, then heated to 80°C at a 5°C per minute ramp, then cooled to 20°C at a 5°C per minute ramp, then held for a 10‐min isothermal hold at 20°C, and finally the samples were heated to 80°C at a 5°C per minute ramp.

Figures 3b and 4b and S4 method

The heating runs consisted of the samples being equilibrated at either 10 or 20°C and then heated to at least 80°C at a 5°C per minute ramp.

### 4.23 Coiled‐coil predictions

Waggawagga web‐based user interface was used to predict coiled‐coil propensity on CAHS D and variants. Waggawagga is designed to provide a direct schematic comparison of many coiled‐coil prediction tools. This interface allows the comparative analysis of six coiled‐coil classic prediction programs (Marcoil, Multicoil, Multicoil2, Ncoils, Paircoil, Paircoil2) and three oligomerization state prediction programs (Scorer, PrOCoil, and LOGICOIL). In addition, Multicoil2 distinguishes dimers, trimers and non‐coiled‐coil oligomerization states. These tools can be run in any combination against single or multiple query sequences (Simm et al., 2015).

For our comparative analysis, we run Marcoil, Multicoil2, Ncoils, and Paircoil2 classic prediction programs on a 14 aa window length with Scorer and LOGICOIL oligomerization state prediction programs.

### 4.24 Crosslinking assays

BS3 (bis[sulfosuccinimidyl] suberate) (Catalog A39266, Thermo Scientific) a 11.4 Å arm amine ester crosslinker was used following manufacturer's instructions in the crosslinking assays.

In Figure 3f–i, a solution of 1 mg/mL of each protein was crosslinked with increasing amounts of BS3 (0.8–8 mM) for 30 min at room temperature. A sample with no crosslinker was used as control for the monomeric state of the proteins.

In Figure S5D, we looked at the oligomeric state after buffer resolvation and after heat resolvation of CAHS D gels and non‐crystalline solids. To obtain a CAHS D gel, a 20 mg/mL gel sample was prepared as described in the temperature and concentration dependent resolubility assay section. To prepare the non‐crystalline solids, a gel formed with 20 mg/mL CAHS D was further dessicated in a speedvac (OFP400, Thermo Fisher Scientific) for 16 h. The crosslinking assay was performed as follows; 2 mg/mL of CAHS D solution was crosslinked with 1 mM BS3 (lane 3) for 30′ at room temperature. As control for monomeric state, a sample of 2 mg/mL CAHS D without crosslinker was used (lane 1). In lane 3, a CAHS D gel formed at 20 mg/mL protein was resolubilized adding buffer to 2 mg/mL concentration and crosslinked with 1 mM BS3 for 30′ at room temperature. In lane 4, the buffer resolubilized gel was further heat resolubilized at 55°C until was clear and then crosslinked with 1 mM BS3 for 30′ at room temperature. In lanes 5 and 6, the same experiment was performed as in lanes 3 and 4 but using CAHS D non‐crystalline solids.

After crosslinking assays, 15 μL of each sample was run in denaturing SDS‐Page gels and stained with Coomassie blue to visualize CAHS D and the variants oligomeric states.

### 4.25 2D‐IR

A detailed description of the setup used to measure the 2D‐IR spectra can be found in (Huerta‐Viga et al., 2010). Briefly, pulses of a wavelength of 800 nm and with a 40 fs duration were generated by a Ti:sapphire oscillator and further amplified by a Ti:sapphire regenerative amplifier to obtain 800 nm pulses at a 1 kHz repetition rate. These pulses were then converted in an optical parametric amplifier to obtain mid‐IR pulses (∼20 μJ, ∼6100 nm) that had a spectral full width at half‐maximum (FWHM) of 150 cm^−1^. The beam was then split into probe and reference beams (each 5%) and a pump beam (90%) that was aligned by a Fabry–Pérot interferometer. The pump and probe beams overlapped in the sample in an ∼250 μm focus. The transmitted spectra of the probe (*T*) and reference (*T*_0_) beams with the pump on and off were then recorded after dispersion by an Oriel MS260i spectrograph (Newport, Irvine, CA) onto a 32‐pixel mercury cadmium telluride (MCT) array. The probe spectrum was normalized to the reference spectrum to compensate for pulse‐to‐pulse energy fluctuations. The 2D‐IR signal was obtained by subtracting the probe absorption in the presence and absence of the pump pulse. Parallel and perpendicular 2D‐IR spectra were recorded by rotating the pump beam at a 45° angle with respect to the probe beam and selecting the probe beam component that was either perpendicular or parallel to the pump beam using a polarizer after the sample. To minimize pump‐scattering contributions, we measured the average of two photoelastic modulator (PEM)‐induced pump delays, such that the interference between the scattered pump beam and the probe beam had a 180° phase in one delay with respect to the other delay.

### 4.26 FTIR analysis of the amide I′ band in CAHS D gels and glasses at increasing hydration

With the aim of assessing CAHS D secondary structure and its possible changes upon vitrification and dehydration we performed a FTIR spectral analysis of CAHS D amide I band in D_2_O (amide I′ band) (Barth, 2007). Any particular secondary structure absorbs predominantly in a specific range of the amide I′ region; due to overlapping, however, the resulting amide I′ band is scarcely structured, and band narrowing procedures are necessary to resolve the various secondary structure components (Barth & Zscherp, 2002). The number and positions of overlapping spectral components have been evaluated from the minima and maxima of the second and fourth derivatives, respectively, of the amide I′ band (Butler & Hopkins, 1970). Second and fourth derivative spectra were calculated using the i‐signal program (version 2.72) included in the SPECTRUM suite (http://terpconnect.umd.edu/~toh/ spectrum/iSignal.html) written in MATLAB language. A Savitsky‐Golay algorithm was employed to smooth the signals and calculate the derivatives. For all the amide I′ bands analyzed, in gel and glassy samples, both the second and fourth derivative spectra were consistently dominated by three minima and maxima, respectively, suggesting the presence of three spectral components, with peak wavenumbers in the intervals 1619–1628 cm^−1^ (ascribed to β–β contacts), 1644–1648 cm^−1^ (unordered/helical regions), and 1676–1688 cm^−1^ (β–β contacts) (Barth, 2007; Barth & Zscherp, 2002). Amide I′ bands were decomposed into the identified three Gaussian components by using a locally developed least‐squares minimization routine (Malferrari et al., 2015) based on a modified grid search algorithm (Bevington & Bevington, 1969). Confidence intervals for the best‐fit parameters were evaluated numerically, as previously detailed (Francia et al., 2009). The fractional area of each component band was taken as the relative content of the corresponding secondary structures (Figure 4e).

### 4.27 Thioflavin T assay

Proteins were dissolved in phosphate buffered saline, pH 7.2 (Sigma‐Aldrich). Thioflavin T was prepared fresh for each assay at a concentration of 400 μM in DMSO, then diluted to 20 μM in PBS/Tris. Twenty‐five microliters of protein and ThT in PBS/Tris were combined in a 96‐well plate (Catalog 3596, Costar, Fisher Scientific) and incubated for 15 min at room temperature in the dark. Fluorescence was measured using a plate reader (Spark 10 M, Tecan) with an excitation at 440 nm, emission was collected at 486 nm. Assays performed using Tris rather than PBS were those measuring CLC due to solubility issues, which was resuspended in 20 mM Tris buffer pH 7.5. For these assays, Thioflavin T was also diluted in this buffer. All proteins and controls were assayed in triplicate.

### 4.28 Cell line generation and maintenance

Full‐length CAHS D (Uniprot P0CU50, CAHS 94063) and CAHS D variants FL_proline and 2X Linker with mVenus proteins fused in their *N*‐termini were cloned into pTwist‐cmv‐WPRE‐Neo between HindIII and BamHI by Twist Bioscience. One microgram of plasmid DNA expressing mVenus:CAHS D, mVenus:FL_ Proline and mVenus:2x Linker proteins were transfected into HEK293 (Catalog CRL‐1573, ATCC) cells with lipofectamine 3000 transfection reagent (Catalog L3000008, Thermo Fisher Scientific). Twenty‐four hours post‐transfection, cells that had successfully integrated mVenus, mVenus:CAHS D, mVenus:FL_Proline and mVenus:2X Linker were selected with 0.7 μg/μL G418 (Catalog G64500, Research products international). Cells were passed twice before expanding and flash‐cooling. Stable cell lines were maintained by supplementing Dulbecco's modified Eagle's medium (DMEM) (Catalog 10567014, Gibco) with 10% fetal bovine serum (FBS) (Catalog 900‐108, GeminiBio Products), 1% penicillin/streptomycin (Catalog 400‐109, Gemini Bio products) and 0.3 μg/μL G418 (Catalog G64500, Research products international) at 37°C with 5% CO_2_ atmosphere.

### 4.29 Fluorescence imaging

8‐well glass bottom dishes (Catalog 80826, Ibidi) were pre‐coated with 0.1 mg/mL poly‐d lysine (Catalog 3890401, Thermo Fisher) for 1 h at 37°C. Cells expressing mVenus, mVenus:CAHS D, mVenus:FL_Proline and mVenus:2X Linker were seeded at a density of 1.0 ×10^5^ cells/mL and allowed to recover overnight. Twenty‐four hours after seeding the cells were stained using imaging medium (FluoroBrite DMEM, Catalog A1896701, Thermo Fisher Scientific) supplemented with 5 μg/mL Hoechst 33342 dye (Catalog 62249, Thermo Fisher) and 1× Cell Brite Red (Catalog 30108‐T, Biotinum) for nucleus and membrane staining. After staining, cells were imaged as non‐stressed in isosmotic imaging medium (FluoroBrite DMEM, Catalog A1896701, Thermo Fisher Scientific) or stressed in hyperosmotic imaging medium (FluoroBrite DMEM, Catalog A1896701, Thermo Fisher Scientific) supplemented with 0.5 M sorbitol.

During the observation, live cells were imaged in a stage incubator with 5% CO_2_ at 37°C.

Images were acquired using a Zeiss 980 Laser Scanning Confocal microscope equipped with a Plan‐Apochromat 63× oil objective, 40× multi‐immersion LD LCI Plan‐Apochromat objective, and a 20× air Plan‐Apochromat objective, LSM 980 (Zeiss Instruments). Data acquisition used ZEN 3.1 Blue software (Zeiss Instruments). Hoechst 33342 dye was excited by the 405 nm laser light with the spectral detector set to 409–481 nm. mVenus protein was excited by the 488 nm laser light with the spectral detector set to 495–550 nm. Cell Brite Red was excited by the 639 nm laser with the spectral detector set to 640–720 nm. Images were processed using ZEN 3.1 Blue software airyscan tool. Data analysis of the Z‐stacks to generate maximum intensity projections was performed in FIJI (V. 2.3.0/1.52q).

### 4.30 Quantification of condensation

Images used for condensate quantification were obtained and processed as detailed above. Images were then loaded into FIJI (V. 2.3.0/1.52q). Color channels were split and the channel corresponding to 488 nm (green) was selected. To mask condensates, thresholding was performed by selecting a range of intensities from 230 to 250. The Analyze Particles tool was used on masked condensates and integrated densities recorded for the entire image.

### 4.31 Cell viability assays

Cells expressing mVenus, mVenus:CAHS D, mVenus:FL_Proline and mVenus:2X Linker were seeded in two sets of triplicates at a density of 1.0 ×10^5^ cells/mL in 96‐well plates (Catalog 3596, Costar, Fisher Scientific).

Twenty‐four hours after seeding, one set of triplicates of each variant was exposed for 24 h to hyperosmotic DMEM media containing 0.5 M sorbitol (stressed cells), while the other set of triplicates was fluid changed with isosmotic DMEM media (non‐stressed control cells). Cells were incubated at 37°C under 5% CO_2_ atmosphere.

After 24 h of hyperosmotic stress each well was stained with 5 μg/mL Hoechst 33342 dye (Catalog 62249, Thermo Fisher), 1 μg/mL Propidium Iodide (PI) (Catalog P3566, Thermo Fisher), and 4 μM Calcein AM (Catalog C1430, Thermo Fisher) for 30 min and their fluorescence measured with a plate reader (Tecan Spark600 Cyto). Cell viability was measured using two methods for the same well, one using Hoechst 33342/PI dye set (Spence & Johnson, 2010; Tanaka et al., 2022) and the other using Calcein AM/PI dye set (Spence & Johnson, 2010). For the Hoescht 33342/PI set measurement, Hoechst 33342‐positive cells and PI‐negative cells were counted as alive cells and Hoechst 33342‐positive and PI‐positive cells were counted as dead cells. For the Calcein AM/PI set measurement, Calcein AM‐positive cells and PI negative cells were counted as alive cells and Calcein AM negative cells and PI‐positive cells were counted as dead cells. The survival rates were calculated by normalizing the cells in stressed wells to the cells in non‐stressed wells.

Measuring the viability of the cells using two different methods gave us a more accurate reading of the cell viability within the wells that help us avoid possible bias introduced using only one method. In all the experiments the normalized survival measured with both methods gave us the same results (Figure S6C–E).

To measure the survival of the cells after 24 h hyperosmotic stress followed by 24 h recovery the same approach was used, but after the 24 h hyperosmotic stress both sets of triplicates were fluid changed to isosmotic DMEM and the cells were left to recover from hyperosmosis at 37°C under 5% CO2 atmosphere for 24 h. After the recovery time, the viability of the cells was measured using both methods as described above.

### 4.32 ER stress and unfolded protein response (UPR) assay

To assess ER stress/UPR, cells were assayed using a Green/Red Ratiometric Cell Stress Assay Kit (Catalog U0901G, Montana Molecular) (Harlen et al., 2019).

Following the commercial kit protocol for adherent cells, cells were seeded at 1.0 ×10^5^ cells/mL in 100 μL of DMEM media in a 96 well plate. Each cell line (naive HEK293, mVenus:CAHS D, mVenus:FL_Proline, mVenus:2X Linker and mVenus) was seeded in triplicate.

For each transduction 10 μL of the Bacman sensor was mixed with 0.3 μL of 500 mM Sodium Butyrate and 39.7 μL of media and added on top of the seeded cells. Cells were incubated at 37°C under 5% CO_2_ atmosphere.

After 4 h incubation time, media was replaced with new media supplemented with 2 mM Sodium Butyrate and cells were further incubated at 37°C under 5% CO_2_ atmosphere for 20 h.

Media was again replaced with fluorobrite imaging media supplemented with 2 mM Sodium Butyrate and red and green fluorescence was measured in a plate reader (Tecan Spark600 Cyto), this measurement was *T* = 0 h. Cells expressing the sensor will have nuclear red fluorescence and cells showing ER stress/UPR will have red and green nuclear fluorescence. Measurements were taken after 6 h (*T* = 6 h) and after 24 h (*T* = 24 h).

Background red and green fluorescence was subtracted from control cells that were not transduced. The normalized fraction of ER stress/UPR was measured dividing the green cell fluorescence by the red cell fluorescence.

### 4.33 Volumetric measurements

Cells were seeded and stained as mentioned in the fluorescence imaging section. Full Z‐stacks of entire cells in isosmotic imaging media or hyperosmotic imaging media were acquired using an LSM 980 (Zeiss Instruments) microscope with ZEN 3.1 Blue software (Zeiss Instruments) in airyscan to perform super resolution imaging. Individual cell volumes of stressed and non‐stressed cells of all the variants were measured using the Imaris Cell Imaging Software (Oxford Instruments). Images from three independent experiments were analyzed and volumes of at least 20 cells per construct and condition were measured for a total of 213 cells. To obtain the relative volume change, the cell volumes of stressed cells were normalized to the non‐stressed cell volumes, and the difference was plotted as % of cell reduction.

### 4.34 Time‐lapse imaging of recovery from hyperosmotic stress

Cell lines expressing mV:CAHS D and mV:2X Linker variants were seeded and stained with 5 μg/mL Hoechst 33342 dye (Catalog 62249, Thermo Fisher) for nuclear staining and 1× Cell Brite Red (Catalog 30108‐T, Biotinum) for membrane staining as mentioned in cell line generation and imaging section. The cells were initially observed in isosmotic imaging medium for ~5 min. Then, 0.5 M sorbitol was added to the imaging medium and the cells were incubated in hyperosmosis for about 10–20 min until clear condensation of the cells was observed. Once condensation was observed, the medium was replaced again with isosmotic imaging medium, and the cells were imaged as a time lapse measuring every 5 s. Images were processed using ZEN 3.1 Blue software airyscan tool and data analysis was performed in FIJI (V. 2.3.0/1.52q).

### 4.35 Measurement of the metabolic rate of the cells

The metabolic activity of the cells was evaluated also using two methods to validate the metabolic rate. In the first method, we measured the resorufin fluorescence using presto blue HS assay (Catalog P50200, Thermo Fisher) (PrestoBlue HS, 2019; Riss et al., 2016). This assay measures the ability of metabolically‐active cells to enzymatically reduce resazurin to resorufin. For the second method, we used Cyquant XTT (Catalog X12223, Thermo Fisher) assay. XTT is a colorimetric assay based on the reduction of a yellow tetrazolium salt (sodium 3′‐[1‐(phenylamino carbonyl)‐3,4‐tetrazolium]‐bis (4‐methoxy6‐nitro) benzene sulfonic acid hydrate) to an orange formazan dye by metabolically active cells. The formazan dye formed is soluble in aqueous solutions and can be directly quantified using a plate reader (Riss et al., 2016; Roehm et al., 1991). Cells expressing mVenus, mVenus:CAHS D, mVenus:FL_Proline and mVenus:2X Linker were seeded in two sets of triplicates at a density of 1.0 ×10^5^ cells/mL in 96‐well plates.

Twenty‐four hours after seeding, one set of triplicates of each variant was exposed for 24 h to hyperosmotic DMEM media containing 0.5 M sorbitol (stressed cells) while the other set of triplicates was fluid changed with isosmotic DMEM media (non‐stressed control cells). Cells were incubated at 37°C under 5% CO_2_ atmosphere.

After 24 h of hyperosmotic stress, either 10 μL/well of Presto blue HS or 70 μL/well of Cyquant XTT working solution was added and the cells were incubated for a fixed time of 4 h in all cases.

Presto Blue's resorufin fluorescence was measured with a plate reader (Tecan Spark600 Cyto) following manufacturer instructions using an excitation/emission wavelength of 560/590 nm (PrestoBlue HS, 2019). Cyquant XTT absorbance was measured following manufacturer instructions at 450 and 660 nm. Blank wells with only media were added for each assay to subtract background signal. Survival by metabolic activity was quantified, normalizing the fluorescence or absorbance of stressed cells with non‐stressed cells.

The same approach was used to measure the survival by metabolic activity of the cells after 24 h hyperosmotic stress followed by 24 h recovery. After the 24 h hyperosmotic stress both sets of triplicates were fluid changed to isosmotic DMEM and cells were left to recover at 37°C under 5% CO2 atmosphere for 24 h. After the recovery time either presto Blue HS or Cyquant XTT were added and the survival by metabolic activity was measured.

To calculate the metabolic rate per live cell (Figure 7e, Figure S6H for Presto Blue, Figure S6I,J for Cyquant XTT), the survival by metabolic activity measured with Presto Blue HS and Cyquant XTT was normalized to the survival measured by the exclusion dyes Hoechst 33342, Calcein AM and PI (Figure S6E).

To compare the metabolic rate to cells that don't express any of our variants, “control cells,” we normalized the metabolic rates of the cells expressing CAHS D and the variants to the metabolic rates of cells expressing mVenus control (Figure 7i for Presto Blue and Figure S6J for Cyquant XTT).

### 4.36 Quantification of cell fluorescence

#### 4.36.1 Microscopy images

Images used for cell fluorescence quantification were obtained and processed as detailed above in the fluorescence imaging section. Images were then loaded into FIJI (V. 2.3.0/1.52q), color channels were split and the channel corresponding to 488 nm (green) was selected. Cells were outlined using the freeform tool and measurements were set for integrated density and mean gray values. Regions of no fluorescence were recorded for each image to use as background.

The corrected total cell fluorescence (CTCF) was calculated using the following formula:

$$\text{CTCF}=\begin{array}{l} \text{Integrated density values}-\left(\text{Area of selected cell}\times \text{Mean fluorescence of background reading}\right). \end{array}$$

#### 4.36.2 Plate reader

Cells expressing mVenus, mVenus:CAHS D, mVenus:FL_Proline and mVenus:2X Linker were seeded at a density of 1.0 ×10^5^ cells/mL in a clear bottom black 96‐well plate (Catalog 3603, Corning) green fluorescence was measured with a plate reader (Tecan Spark600 Cyto) using the cell imaging mode with the green filter sets.

### 4.37 Quantification and statistical analysis

All‐atom simulations were run using the ABSINTH implicit solvent model and CAMPARI Monte Carlo simulation engine (https://campari.sourceforge.net/). Simulations were analyzed using SOURSOP (https://github.com/holehouse-lab/soursop) and MDTraj. IDR kappa values were calculated using sparrow.

SAXS data were reduced at the beamline using the BioXTAS RAW 1.4.0 software (Hopkins et al., 2017). Buffer subtraction, subsequent Guinier fits, and Kratky transformations were done using custom MATLAB (Mathworks, Portola Valley, CA) scripts.

FTIR data were analyzed using the i‐signal program (version 2.72) included in the SPECTRUM suite (http://terpconnect.umd.edu/~toh/spectrum/iSignal.html) written in MATLAB language.

The statistical analysis of cell assays was performed using Microsoft Excel (v.2201, build 14827.20198) and R studio (R v4.1.0, R studio v 1.4.1717; packages ggplot2, gridExtra, readxl, dplyr, ggsignif). The statistical details of each experiment can be found embedded in the results section and in the figure legends, including the type of statistical test used, and significance thresholds.

Correlation plots were analyzed by generating a Pearson correlation coefficient in R Studio (R v 4.2.3, R studio v 2023.03.0 + 386). The subsequent *p*‐value was used to assess the significance of each correlation.

## AUTHOR CONTRIBUTIONS

**T. C. Boothby:** Conceptualization; funding acquisition; writing – original draft; writing – review and editing; supervision; project administration. **S. Sanchez‐Martinez:** Conceptualization; investigation; writing – original draft; writing – review and editing; methodology; validation; visualization; formal analysis; data curation; supervision. **K. Nguyen:** Investigation; writing – original draft; writing – review and editing; methodology; formal analysis; data curation; visualization. **S. Biswas:** Investigation; writing – original draft; writing – review and editing; formal analysis; methodology; data curation; visualization. **V. Nicholson:** Investigation; writing – original draft; data curation; formal analysis; visualization. **A. V. Romanyuk:** Investigation; writing – review and editing; writing – original draft; formal analysis; data curation; methodology; visualization. **J. Ramirez:** Investigation; writing – original draft; writing – review and editing; formal analysis; methodology; data curation; visualization. **S. Kc:** Investigation; writing – original draft; writing – review and editing; methodology; formal analysis; data curation; visualization. **A. Akter:** Investigation; writing – review and editing; visualization. **C. Childs:** Investigation; writing – review and editing. **E. K. Meese:** Investigation; writing – review and editing; visualization. **E. T. Usher:** Investigation; writing – review and editing. **G. M. Ginell:** Investigation; writing – review and editing. **F. Yu:** Investigation; writing – review and editing. **E. Gollub:** Investigation; writing – review and editing. **M. Malferrari:** Investigation; writing – review and editing; methodology; formal analysis; data curation. **F. Francia:** Investigation; writing – review and editing; formal analysis; data curation; methodology. **G. Venturoli:** Investigation; writing – original draft; methodology; formal analysis; data curation. **E. W. Martin:** Investigation; writing – review and editing; visualization; formal analysis. **F. Caporaletti:** Investigation; writing – review and editing; methodology; formal analysis; data curation. **G. Giubertoni:** Investigation; writing – review and editing; methodology; data curation; formal analysis. **S. Woutersen:** Writing – review and editing; supervision. **S. Sukenik:** Writing – review and editing; supervision. **D. N. Woolfson:** Writing – review and editing; supervision. **A. S. Holehouse:** Writing – review and editing; supervision.

## CONFLICT OF INTEREST STATEMENT

The authors declare no competing interests.

## Supporting information


**Data S1.** Supporting Information.
