# Supplementary material for: Labile assembly of a tardigrade protein induces biostasis
Source: Protein Sci. 2024 Mar 19;33(4):e4941. doi: 10.1002/pro.4941 (PMC10949331; doi:10.1002/pro.4941)
Supplement: Supplementary file 1 — Data S1. Supporting Information. [file PRO-33-e4941-s001.docx]

**Title:** Labile assembly of a tardigrade protein induces biostasis

**Authors:** Sanchez-Martinez S.^1^, Nguyen K.^1^, Biswas S.^1^, Nicholson V.^1^, Romanyuk A.V.^2,3^, Ramirez J.^1^, KC S.^1^, Akter A.^1^, Childs C.^1^, Meese, E.K.^1^, Usher E.T.^4,5^, Ginell G.M.^4,5^, Yu F.^6^, Gollub E.^7^, Malferrari M.^8^, Francia F.^9^, Venturoli G.^9,10^, Martin E.W.^11^, Caporaletti F.^12^, Giubertoni G.^12^, Woutersen S.^12^, Sukenik S.^6,7^, Woolfson D.N.^2,3,13^, Holehouse A.S.^4,5^, Boothby T.C.^1,*^

**Supplementary Text and Figures**

To determine what ensemble structure within the termini drives gel formation, we performed femtosecond two-dimensional infrared (2D-IR) spectroscopy on CAHS D solutions at concentrations below and above the critical concentration for gelation. The amide I vibration of the backbone amide groups is particularly sensitive to the protein conformation [(Barth & Zscherp, 2002)](https://paperpile.com/c/SdFjWL/Irk0E). In particular, in β-sheet and α-helix structures, the spatial arrangement of the amide groups causes excitonic couplings between the amide I vibrations, thus giving rise to delocalized normal modes, which in the case of β-sheet absorbs at 1620–1630 and 1680–1700 cm^–1^ [(Barth & Zscherp, 2002)](https://paperpile.com/c/SdFjWL/Irk0E). 2D-IR Spectroscopy detects the couplings between these normal modes, which appear as off-diagonal features (cross peaks) in the two-dimensional spectrum [(Hamm & Zanni, 2011)](https://paperpile.com/c/SdFjWL/EPKQl). These 2D-IR cross peaks are reliable markers for the presence of β-sheet structures [(Cheatum et al., 2004; Demirdöven et al., 2004)](https://paperpile.com/c/SdFjWL/nRjVB+wjAa1), in particular in the case where β-sheet features may be difficult to discern in the conventional FTIR spectrum, for instance due to the presence of other secondary structures in the protein [(Giubertoni et al., 2022)](https://paperpile.com/c/SdFjWL/SN930)

Figure 4C shows the 2D-IR spectrum of CAHS D above the gelation threshold (2.5 wt%). We observe two intense diagonal peaks at pump frequencies of 1630 and 1650 cm^-1^, and a weaker one at 1690 cm^-1^. When exciting at the pump frequency of 1630 cm^-1^, we observe a cross-peak signature at a probe frequency of 1690 cm^-1^, which is a marker of β-sheet structure [(Cheatum et al., 2004; Giubertoni et al., 2022)](https://paperpile.com/c/SdFjWL/wjAa1+SN930). Figure 4D shows a horizontal slice through the 2D-IR spectrum (obtained by averaging over the pump-frequency range from 1625 to 1635 cm^-1^) in which the cross peak is better visible. The cross peak is absent in the 2D-IR signal when measuring at a CAHS D concentration of 0.5 wt% (Fig. 4D), indicating that CAHS D only adopts a β-sheet conformation when the concentration is above the critical concentration for gelation.

We then assessed how the conformation of CAHS D changes in going from the gelled to the desiccated state. To this end, we performed FTIR experiments on dried CAHS D gels by fitting the infrared spectra using three Gaussian-shaped bands in agreement with the 2D-IR results. We observed that β-sheet content increases in the hydrated state as a function of concentration (Fig. 4D). This increase continues in the drying hydrogel (100-95% relative humidity) but then begins to decrease at lower hydration levels (75-11% relative humidity) (Fig. 4E). This implies that there is an optimal hydration level for stabilizing β-β contacts, which may relate to the need for higher stability while the matrix is undergoing the final stages of drying or the early stages of rehydration.

**
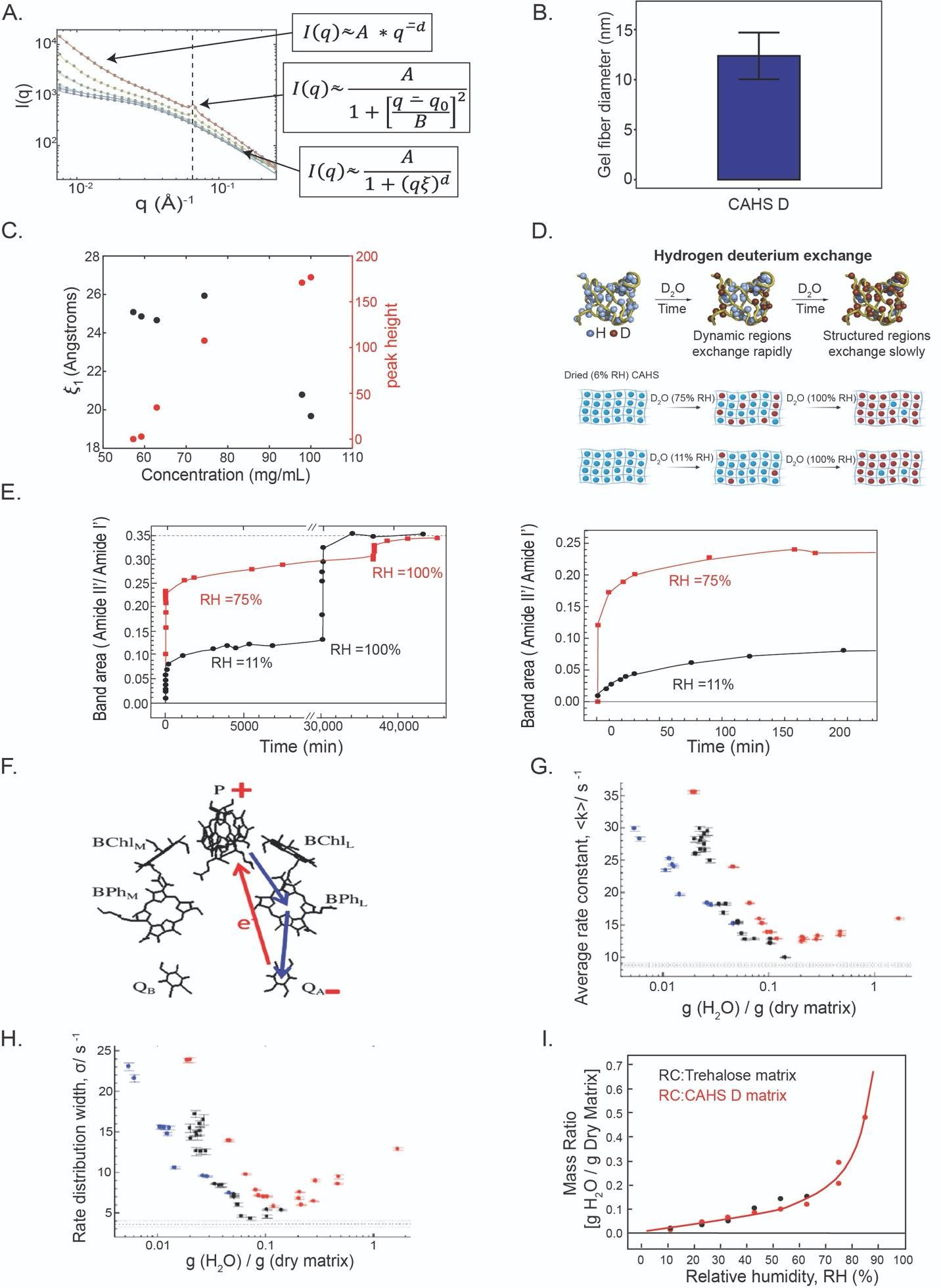
Supplementary Figure 1 (Related to Figure 1). A)** Concentration gradient SAXS on CAHS D and associated equations. **B)** Quantification of gel fiber diameter from SEM images of CAHS D (n = 20). **C)** SAXS analysis and quantification of void space sizing within a CAHS D fiber as a function of concentration of CAHS D. **D)** Schematic representation of hydrogen deuterium exchange experiment. **E)** Hydrogen deuterium exchange results for full time range (left) and for just the initial 200 minutes (right). **F)** Schematic representation of the cofactors of the bacterial photoreactive center used, involved in photoinduced electron transfer and charge recombination. **G)** Average rate constant <k> and **H)** rate distribution width σ of charge recombination kinetics of the photoreactive center (see eq. 1) as a function of hydration level for the reaction center alone (blue), reaction center in a trehalose glass (black), or the reaction center in a CAHS D gel (red). Dashed horizontal lines correspond to <k> and σ values determined in solution. **I)** Mass ratio of water to dry matrix as a function of relative humidity for a sample composed of trehalose (black) or CAHS D (red) - note both glassy matrices contain similar degrees of water content.

**
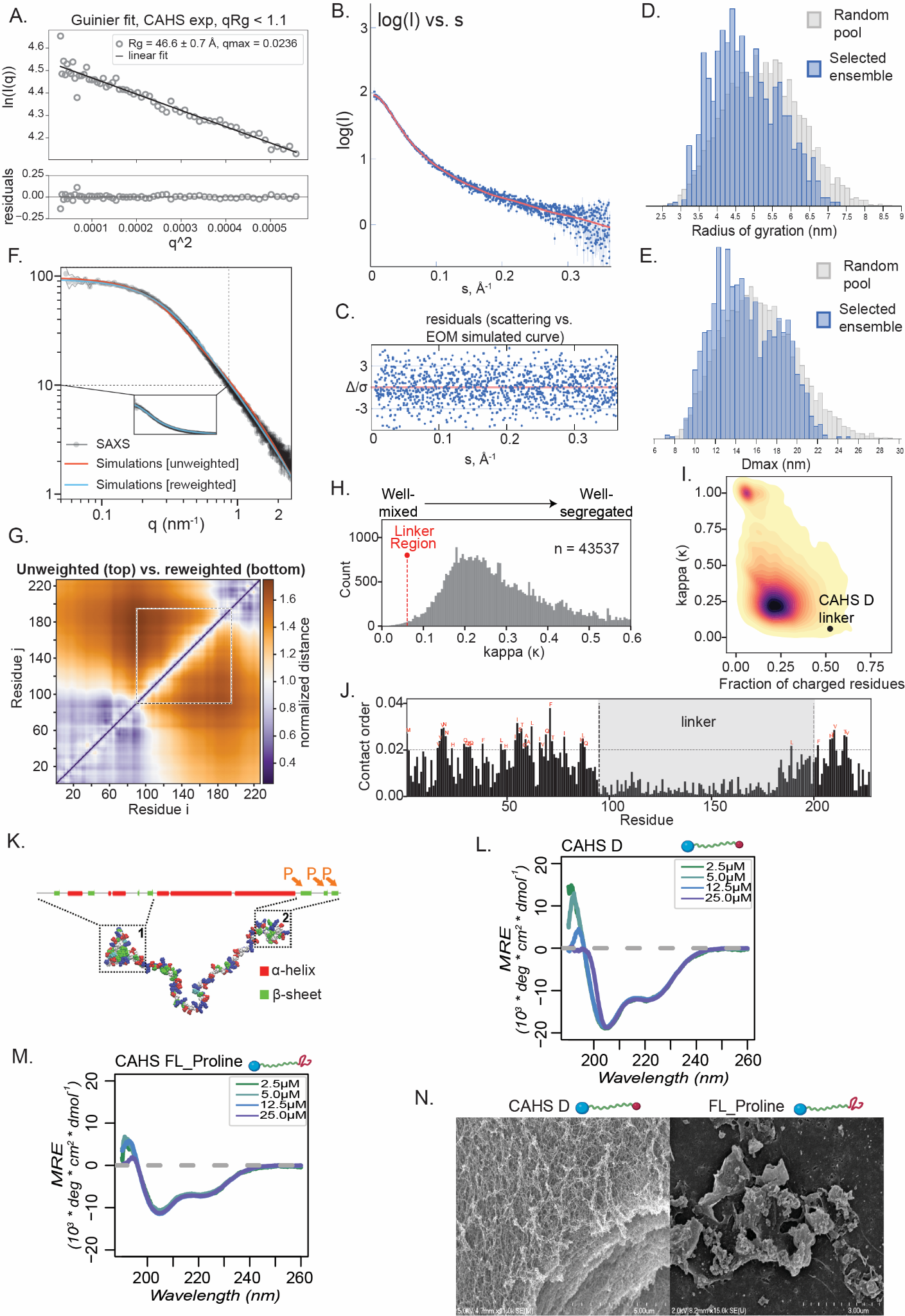
Supplementary Figure 2 (Related to Figure 2). A)** Guinier analysis of scattering data with residuals shown below. **B)** EOM-derived scattering profile compared to experimental data in log(I) vs. s space. **C)** Normalized distribution of experimental SAXS data points around the EOM-derived curve shown in panel B. **D)** Frequency of R_g_ values observed in the initial pool of structures generated by EOM and in subset of structures that were selected by EOM’s genetic algorithm to fit our experimental SAXS data. **E)** Frequency of D_Max_ values observed in random CAHS D structures and in the ensemble selected by EOM. **F)** Comparison of scattering profiles derived from re-weighted simulation ensemble vs. unweighted simulation ensemble. The difference in overall radius of gyration upon reweighting changes from 5.15 nm to 4.88 nm, suggesting this is a small change, in line with the modest changes observed with the scattering profile. **G)** Comparison of inter-residue normalized distance map for unweighted (top left) and re-weighted (bottom right) simulated ensembles confirms no major changes in intramolecular interactions occur upon reweighting. **H)** Plot showing the distribution of kappa values within all IDRs in the *H. exemplaris* proteome with the CAHS D linker annotated as being in the top 1% most well-mixed IDRs. **I)** Kernel density plot highlighting the distribution of IDRs in the *H. exemplaris* proteome in terms of fraction of charge residues and charge patterning. The CAHS D linker is highlighted as an extreme outlier in both dimensions. **J)** Contact order analysis of residues. Residues with a contact order score of 0.02 or higher are named. **K)** Schematic CAHS D with predicted secondary structure and location of Full-Length Proline (FL_Proline) prolines highlighted. **L)** Circular dichroism spectroscopy of CAHS D as a function of concentration. **M)** Circular dichroism spectroscopy of FL_Proline as a function of concentration. **N)** SEM images of FL-Proline at 50 g/L compared to CAHS D gel.

**
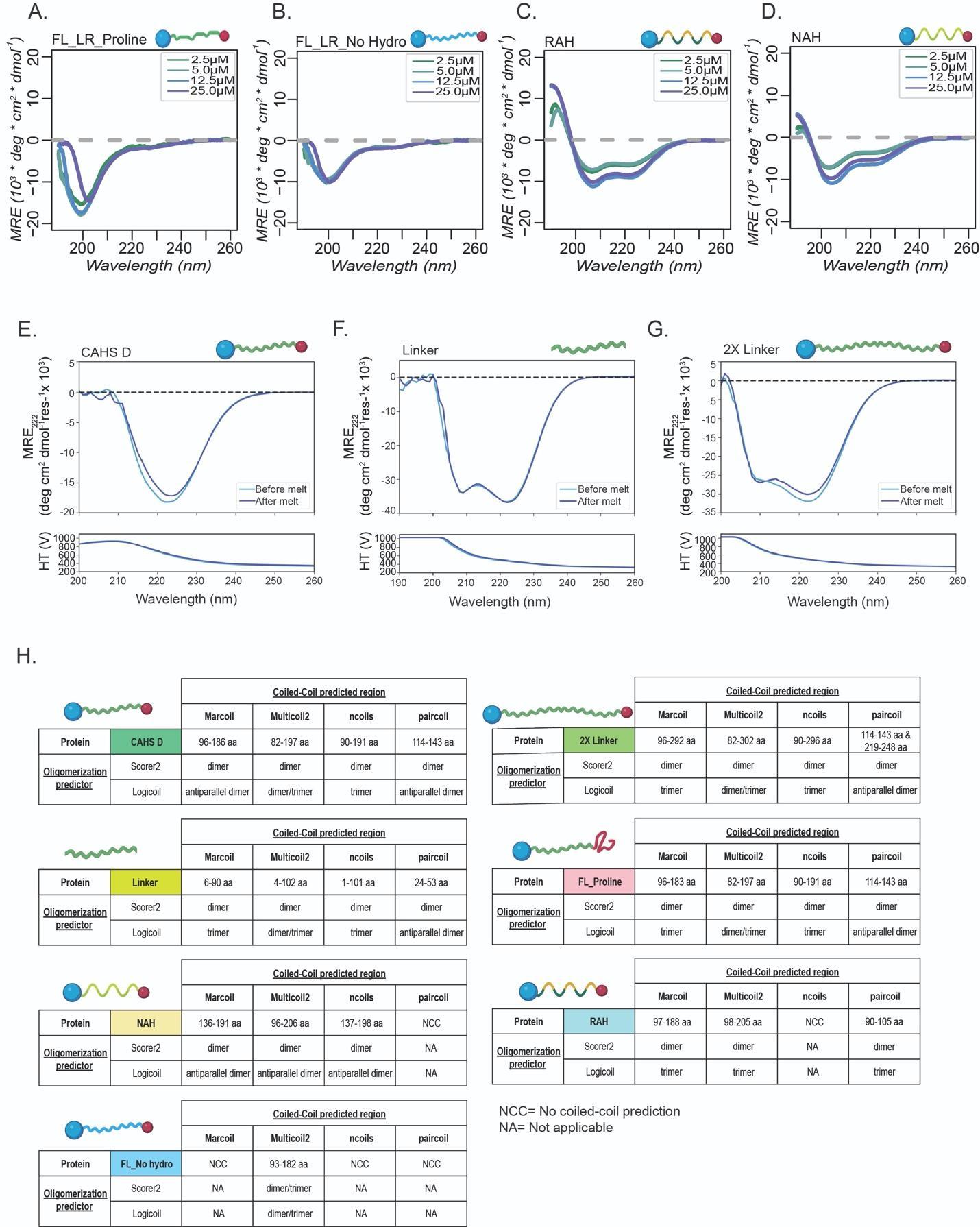
Supplementary Figure 3 (Related to Figure 3). A)** Circular dichroism spectroscopy as a function of concentration of FL_LR Proline, **B)** FL_LR No hydro, **C)** RAH and **D)** NAH variants **E)** CD spectra before (light blue line) and after (dark blue line) thermal denaturation of gelled CAHS D (0.7 mM, 17.7 mg/mL), **F)** LR variant (0.7 mM, 8.6 mg/mL) and **G)** Gelled 2X LR variant (0.25 mM, 9.3 mg/mL). **H)** Coiled-coil predictions for linker variants.

**
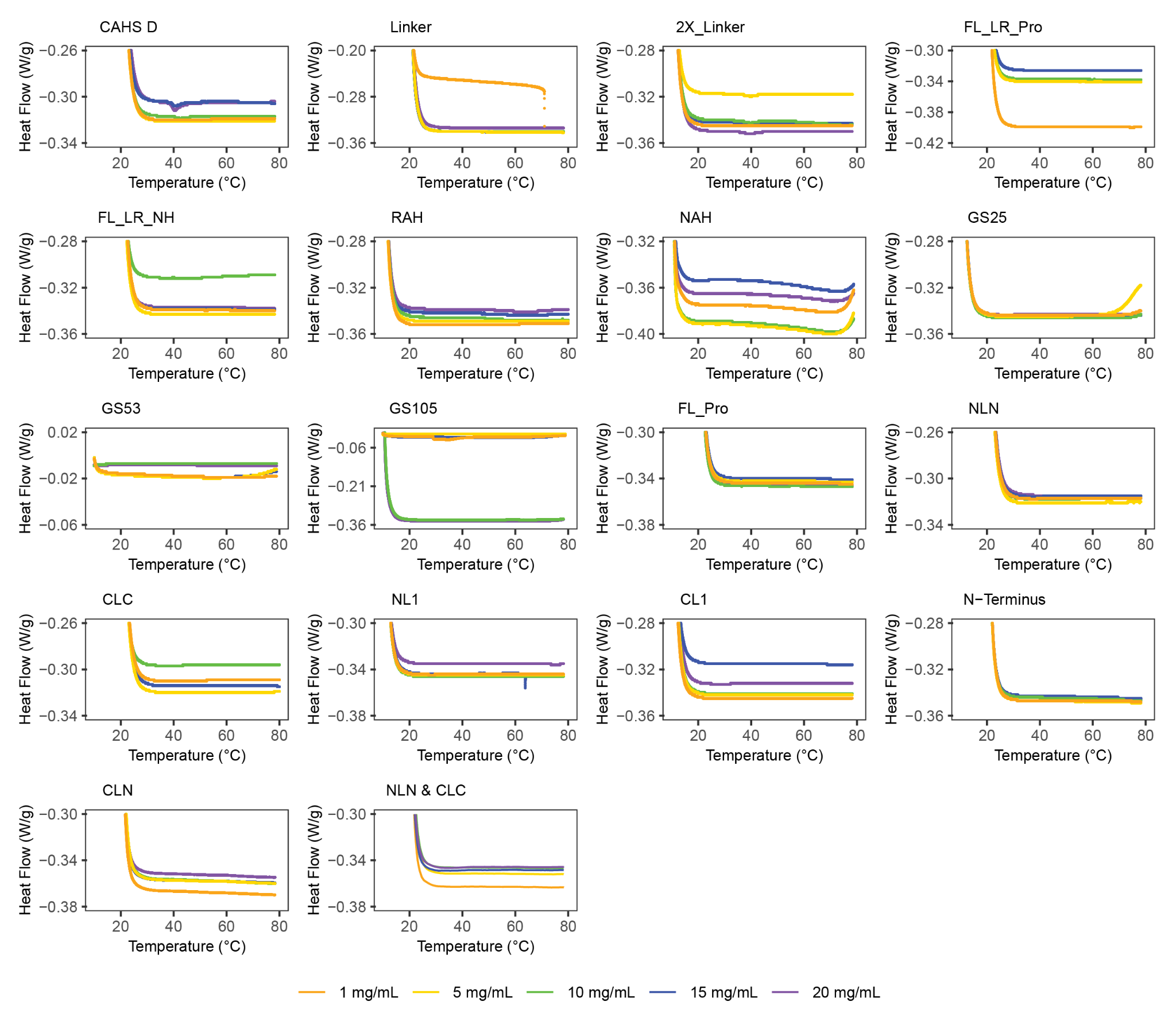
Supplementary Figure 4 (Related to Figure 4). A)** Differential scanning calorimetry thermogram displaying melt curves for CAHS D and its variants at different concentrations.

**
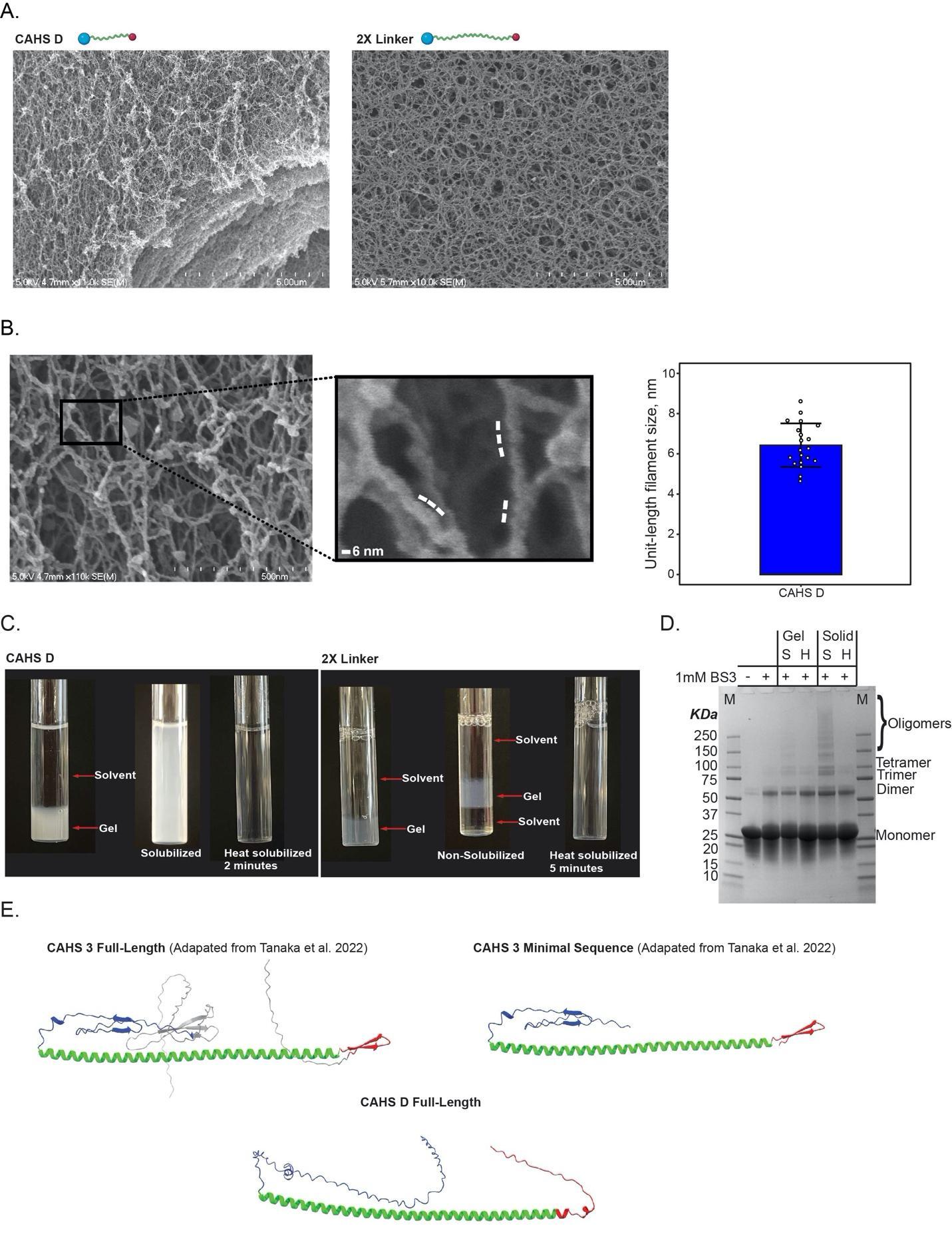
Supplementary Figure 5 (Related to Figure 5). A)** Representative SEM images of CAHS D and 2X Linker gels (50 g/L). **B)** Quantification of “bumps” (Unit-length filaments) on CAHS D fibers. **C)** Dilution of 10 g/L (0.4 mM) CAHS D gel (left) and 2X Linker gel (right) in 20 mM Tris buffer results in instant gel resolvation for CAHS D but no gel resolvation for 2X Linker. Heat resolvation at 55°C happens within ~2 min for CAHS D and ~ 5 min for 2X Linker. **D)** SDS-Page showing resolvation of crosslinked CAHS D gels and solids. First lane shows MW marker. Second lane has 2 mg/mL CAHS D without crosslinker showing monomeric state. Third lane has 2 mg/mL CAHS D crosslinked with 1 mM BS3 showing formation of mostly dimers. Fourth lane shows resolvated CAHS D gel with buffer, down to 2 mg/mL concentration, crosslinked with 1 mM BS3, showing dimers and high order oligomers. Fourth lane is identical to third lane but the gel has been further heat resolubilized prior crosslinking, showing the disappearance of the high order oligomers mirroring the clear solution we see after 55°C heating in Fig. S5C. Lanes five and six are the same as third and fourth but starting the resolvation from a gel that has been dessicated in a speedvac for 16h to turn it into a non-crystalline solid. As in the third lane, after solubilizing with buffer there are still high order oligomers that disappear after heating (lane six). **E)** Alphafold2 predictions of *R. varieornatus* proteins CAHS 3 and the Minimal Sequence of CAHS 3 needed for gelation (Adapted from Tanaka et al. 2022) in comparison with CAHS D protein. Note that the Minimal Sequence of CAHS 3 shows a similar dumbbell-like ensemble as CAHS D. Colored in blue the N-terminal domains (Region 3), in green the Linker domains (Coiled-coil domain) and in red the C-terminal domains (CR2).

**
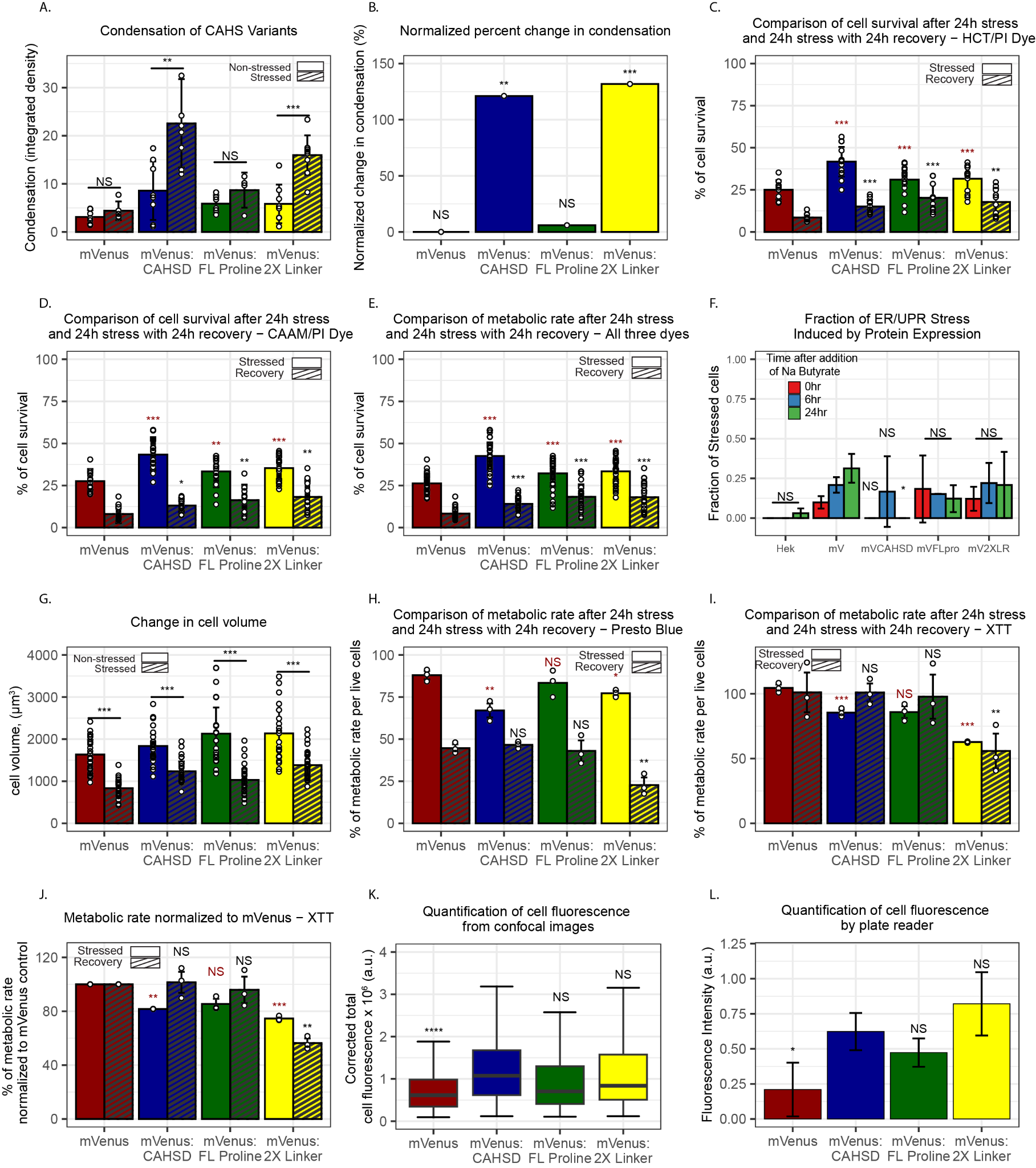
Supplementary Figure 6 (Related to Figure 7).** **A)** Quantification of condensation of CAHS D and its variants before (solid bars) and during osmotic shock (striped bars). **B)** Quantification of change in condensation for CAHS D and its variants during osmotic stress. **C)** Quantification of cell viability during osmotic stress (solid bars) and after recovery (striped bars) using the Hoechst/Propidium iodide assay. **D)** Quantification of cell viability during osmotic stress (solid bars) and after recovery (striped bars) using the Calcein AM/Propidium iodide assay. **E)** Quantification of combined data from Calcein AM/Propidium iodide and Hoechst/Propidium iodide viability assays during osmotic stress (solid bars) and after recovery (striped bars). **F)** ER stress and unfolded protein response from HEK cells expressing mVenus:CAHS D, mVenus:FL_Proline and mVenus:2X Linker compared to overexpressing mVenus protein and naive HEK cells. **G)** Quantification of cell volume before (solid bars) and during osmotic stress (striped bars) in cells expressing CAHS D or its variants. **H)** Quantification of metabolic rates of alive cells during (solid bars) and after (striped bars) osmotic stress using the Presto Blue HS assay. **I)** Quantification of metabolic rates of alive cells during (solid bars) and after (striped bars) osmotic stress using the XTT assay. **J)** Normalized metabolic rates of alive cells to mVenus control cells during osmotic stress (solid bars) and after recovery (striped bars) using the XTT assay. **K)** Quantification of corrected total cell green fluorescence of the different mVenus constructs from microscopy images. **L)**Sum of green fluorescence intensity from cells expressing the different mVenus constructs measured in a plate reader. Error bars represent average deviation. Significance determined using a paired T. Test. Asterisks represent significance relative to non-stressed cells in figures A and G. In Figure B and F asterisks represent significance to mVenus expressing cells. In Figure K and L asterisks represent significance to mVenus-CAHS D. In figures C-E and G-I red color statistics represent significance relative to mVenus stressed cells, and black color statistics represent significance to mVenus recovered cells. *p<0.05, **p<0.01, ***p<0.005, NS is not significant.

[Barth, A., & Zscherp, C. (2002). What vibrations tell us about proteins. *Quarterly Reviews of Biophysics*, *35*(4), 369–430.](http://paperpile.com/b/SdFjWL/Irk0E)

[Cheatum, C. M., Tokmakoff, A., & Knoester, J. (2004). Signatures of beta-sheet secondary structures in linear and two-dimensional infrared spectroscopy. *The Journal of Chemical Physics*, *120*(17), 8201–8215.](http://paperpile.com/b/SdFjWL/wjAa1)

[Demirdöven, N., Cheatum, C. M., Chung, H. S., Khalil, M., Knoester, J., & Tokmakoff, A. (2004). Two-Dimensional Infrared Spectroscopy of Antiparallel β-Sheet Secondary Structure. *Journal of the American Chemical Society*, *126*(25), 7981–7990.](http://paperpile.com/b/SdFjWL/nRjVB)

[Giubertoni, G., Caporaletti, F., Roeters, S. J., Chatterley, A. S., Weidner, T., Laity, P., Holland, C., & Woutersen, S. (2022). In Situ Identification of Secondary Structures in Unpurified Bombyx mori Silk Fibrils Using Polarized Two-Dimensional Infrared Spectroscopy. *Biomacromolecules*, *23*(12), 5340–5349.](http://paperpile.com/b/SdFjWL/SN930)

[Hamm, P., & Zanni, M. (2011). *Concepts and Methods of 2D Infrared Spectroscopy*. Cambridge University Press.](http://paperpile.com/b/SdFjWL/EPKQl)
